# *C. elegans* Clarinet/CLA-1 recruits RIMB-1/RIM-binding protein and UNC-13 to orchestrate presynaptic neurotransmitter release

**DOI:** 10.1073/pnas.2220856120

**Published:** 2023-05-15

**Authors:** Mia Krout, Kelly H. Oh, Ame Xiong, Elisa B. Frankel, Peri T. Kurshan, Hongkyun Kim, Janet E. Richmond

**Affiliations:** ^a^Department of Biological Sciences, University of Illinois at Chicago, Chicago, IL 60607; ^b^Department of Cell Biology and Anatomy, Center for Cancer Cell Biology, Immunology, and Infection, Chicago Medical School, School of Graduate and Postdoctoral Studies, Rosalind Franklin University of Medicine and Science, North Chicago, IL 60064; ^c^Department of Genetics, Albert Einstein College of Medicine, New York, NY 10461

**Keywords:** synaptic transmission, exocytosis, neuromuscular junction, cytomatrix of the active zone, synaptic proteins

## Abstract

Synaptic transmission requires the coordinated activity of multiple synaptic proteins that are localized at the active zone (AZ). We previously identified a *Caenorhabditis elegans* protein named Clarinet (CLA-1) based on homology to the AZ proteins Piccolo, Rab3-interactingmolecule (RIM)/UNC-10 and Fife. At the neuromuscular junction (NMJ), *cla-1 null* mutants exhibit release defects that are greatly exacerbated in *cla-1;unc-10 double* mutants. To gain insights into the coordinated roles of CLA-1 and UNC-10, we examined the relative contributions of each to the function and organization of the AZ. Using a combination of electrophysiology, electron microscopy, and quantitative fluorescence imaging we explored the functional relationship of CLA-1 to other key AZ proteins including: RIM1, Cav2.1 channels, RIM1-binding protein, and Munc13 (*C. elegans* UNC-10, UNC-2, RIMB-1 and UNC-13, respectively). Our analyses show that CLA-1 acts in concert with UNC-10 to regulate UNC-2 calcium channel levels at the synapse via recruitment of RIMB-1. In addition, CLA-1 exerts a RIMB-1-independent role in the localization of the priming factor UNC-13. Thus *C. elegans* CLA-1/UNC-10 exhibit combinatorial effects that have overlapping design principles with other model organisms: RIM/RBP and RIM/ELKS in mouse and Fife/RIM and BRP/RBP in *Drosophila*. These data support a semiconserved arrangement of AZ scaffolding proteins that are necessary for the localization and activation of the fusion machinery within nanodomains for precise coupling to Ca^2+^ channels.

## Significance

*Caenorhabditis elegans* is a round worm that has been successfully used to study synaptic transmission, the process by which neurons communicate with targets cells in the nervous system through the release of chemical neurotransmitters. Many synaptic proteins involved in this release process are highly conserved with some species-specific variations in individual components. Here, we examined how the *C. elegans* protein, clarinet (CLA-1), an elusive member of the Piccolo, Fife and Rab3-interactingmolecule (Rim) protein family, fits into the organization and function of synaptic release sites in the worm. Using CRISPR/Cas-9 to endogenously tag key synaptic players, we determined the functional hierarchy of these *C. elegans* synaptic proteins and provide insights into conserved design principles that govern nervous system function.

Efficient release of neurotransmitters is critical for information exchange between neurons and their targets that govern cognitive and behavioral processes. Neurotransmission is initiated by voltage-dependent Ca^2+^ influx, which triggers the fusion of primed synaptic vesicles (SVs) at functional domains termed active zones (AZs). Although synapses vary in size and morphology, all AZs have electron-dense projections (DPs) packed with specialized proteins that recruit and prime SVs in close proximity to calcium channels. The probability of release has been shown to scale with the size of the AZ underscoring the importance of this proteinaceous scaffold for synaptic function (1). As in other organisms, *Caenorhabditis elegans* AZs contain a cytomatrix of conserved multi-domain proteins, including: synapse defective (SYD-2)/Liprin-α, uncoordinated-10 (UNC-10)/Rab3-interacting molecule (RIM), RIMB-1/RIM-binding protein (RIM-BP), ELKS-1/ELKS (protein rich in the amino acids E, L, K, and S), UNC-13/Munc13, and UNC-2/CaV2 channels (2–6). Functional interactions between these proteins orchestrate the docking and priming of SVs, mediate calcium-channel localization and function, and regulate the probability and timing of neurotransmitter release. Other AZ proteins appear to be more species-specific including mammalian Piccolo and Bassoon and fly Bruchpilot and Fife, but even these proteins exhibit domain conservation and appear to have overlapping functions. Exactly how these proteins contribute to the AZ organization and release characteristics in different organisms remains to be fully elucidated.

In a previous study, we described a novel *C. elegans* AZ protein, named Clarinet (CLA-1) that has homology to Piccolo, RIM and Fife (7). The *cla-1* gene was predicted to encode three isoforms based on size: long(L), medium(M), and short(S). Since the number and distribution of RNAseq splice junctions on Wormbase suggest there are only L- and S-isoforms, the M-isoform was not further explored in this study. CLA-1 L- and S- isoforms share and identical C-terminal PDZ and C2 containing domain, in common with Piccolo, Fife and RIM, which tethers CLA-1 at the AZ (7). The N terminus of the CLA-1 short (CLA-1S) isoform also colocalizes with these AZ markers, while the N terminus of the 9,000 amino acid CLA-1L isoform extends well into the periactive zone. At the neuromuscular junction (NMJ) *cla-1 null* mutants exhibit morphological abnormalities including reduced SV number and DP size and display lower endogenous release rates and increased synaptic depression (7). Importantly, *cla-1* genetically interacts with *unc-10,* the double mutants producing synergistic effects resulting in the complete loss of evoked release (7).

To gain mechanistic insights into the role of CLA-1, we examined the relative contributions of CLA-1 isoforms to the function and organization of the AZ and explored the relationship between CLA-1 and UNC-10. In this study, we show that loss of CLA-1 reduces endogenous RIMB-1 levels, resulting in an exacerbated UNC-2 phenotype in a *cla-1;unc-10* double mutant. Moreover, we demonstrate that CLA-1L and UNC-10 contribute to the synaptic localization of UNC-13. These data provide an understanding of the combined roles of CLA-1 and UNC-10 in synaptic function, revealing why loss of these two proteins together results in the elimination of neurotransmitter release at *C. elegans* NMJs.

## Results

### CLA-1 and UNC-10 Functionally Interact to Support Neuromuscular Activity.

**Fig. 1.**
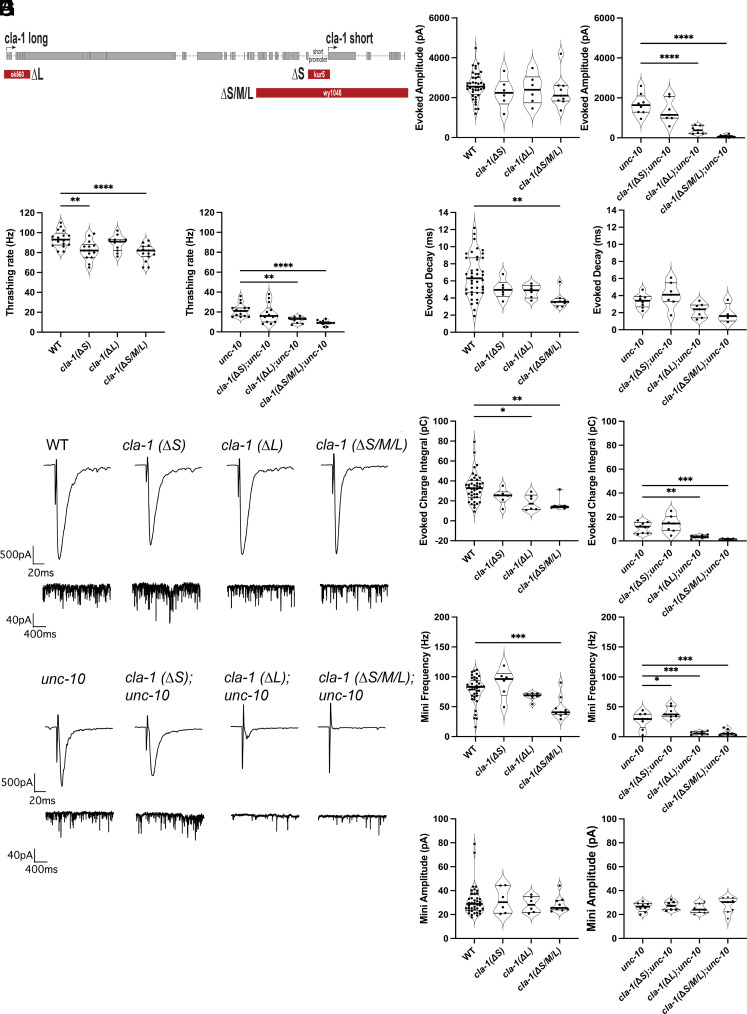
Behavioral and electrophysiology data show further reduction in *cla-1(ΔS/M/L)* and *cla-1(ΔL)* in an *unc-10* mutant background. (*A*) Full-length CLA-1 with allele designations and areas of the deletions for mutants used in this study. (*B*) Though the effect is mild, thrashing rates are significantly reduced in *cla-1(ΔS/M/L)* (*P* < 0.0001) and *cla-1(ΔS)* (*P* < 0.01). In an *unc-10* background both *cla-1(ΔS/M/L)* (*P* < 0.0001) and *cla-1(ΔL)* (*P* < 0.01) are additively reduced beyond *unc-10* mutants alone, whereas *cla-1(ΔS)* mutants were not. (*C*–*H*) In 5 Ca^2+^ ringer none of the *cla-1* single mutants exhibit a change in evoked amplitude; however, both *cla-1(ΔS/M/L)* (*P* < 0.01) and *cla-1(ΔL)* (*P* < 0.05) exhibit a reduction in evoked charged integral. The mini frequency is only reduced in *cla-1(ΔS/M/L)* (*P* < 0.001). However, in an *unc-10* mutant background we see a dramatic defect in evoked amplitude and integral in both the *cla-1(ΔS/M/L)* (amp *P* < 0.0001; int *P* < 0.001) and *cla-1(ΔL)* (amp *P* < 0.0001; int *P* < 0.001). The *cla-1(ΔS/M/L);unc-10* (*P* < 0.001) and *cla-1(ΔL);unc-10* (*P* < 0.001) double mutants have a significant reduction in mini frequency as compared to the *unc-10* mutant, and surprisingly the *cla-1(ΔS);unc-10* double mutants exhibit a slight but significant increase (*P* < 0.05).

To better understand CLA-1 function, we compared the phenotypes of isoform-specific *cla-1* mutants to the null allele *cla-1(wy1048)* (termed *cla-1ΔS/M/L*). For the current study we generated a CLA-1S mutant, *cla-1(kur5),* using Clustered Regularly Interspaced Palindromic Repeats/CRISPR-associated endonuclease (CRISPR/Cas9) (termed *cla-1ΔS*) and used a previously isolated mutant *cla-1(ok560)*, which deletes CLA-1 long (CLA-1L) (termed *cla-1ΔL)* (Fig. 1*A*). After the *cla-1ΔS* deletion was verified (S1), we undertook a characterization of the *cla-1(ΔL)* and *cla-1(ΔS)* mutants and compared their phenotypes to the *cla-1 null (cla-1ΔS/M/L)*.

Mutants affecting synaptic function often display behavioral deficits. Therefore, as an initial screen of the isoform-specific mutants, we examined mobility by scoring worm thrashing rates in solution. We found only slight reductions in thrashing rates in the *cla-1* mutants, which only reached significance in the *cla-1(ΔS/M/L)* and *cla-1(ΔS)* (Fig. 1*B*). Given that *cla-1(ΔS/M/L)* exacerbates the synaptic defect of *unc-10* mutants, we next performed thrashing assays on each of the *cla-1* mutants in combination with the *unc-10* null (7). As expected, based on previously characterized release defects, the thrashing rate of *unc-10* single mutants was reduced to about 25% of the wild type (Fig. 1*B*). In the *unc-10* sensitized background, both the *cla-1(ΔS/M/L);unc-10* and *cla-1(ΔL);unc-10* double mutants exhibited more severe thrashing defects than *unc-10* alone, whereas *cla-1(ΔS);unc-10* mutants did not (Fig. 1*B*).

To determine whether the behavioral phenotypes were recapitulated at the level of the NMJ, we recorded evoked and endogenous release in dissected *cla-1* mutants. As we previously reported, the evoked amplitude in *cla-1(ΔS/M/L)* was not different from the wild type (Fig. 1 *C* and *D*) (7). Similarly, neither *cla-1(ΔL)* nor *cla-1(ΔS)* exhibited reductions in evoked amplitude (Fig. 1 *C* and *D*). However, analysis of the evoked charge integral revealed a significant decrease in *cla-1(ΔS/M/L)* and to a lesser extent the *cla-1(ΔL)* but not the *cla-1(ΔS*) mutants (Fig. 1 *C* and *F*). Of the three *cla-1* mutants, the endogenous mini frequency was only significantly reduced in *cla-1(ΔS/M/L)*, though the long and short mutants trended lower (Fig. 1 *C* and *G*). Consistent with the behavioral phenotypes, when we looked at *cla-1* mutants in the *unc-10* background we saw that both *cla-1(ΔS/M/L) and cla-1(ΔL)* mutants significantly exacerbated the evoked and endogenous synaptic defects, whereas release in *cla-1(ΔS);unc-10* double mutants was similar to *unc-10* (Fig. 1 *C*–*G*). Mini amplitudes were unchanged in any of the mutants suggesting the defects were presynaptic (Fig. 1 *C* and *H*). Together these functional data suggest that the genetic interaction of *cla-1* with *unc-10* primarily reflects contributions of CLA-1L, although as the null allele is more severe than the *cla-1(ΔL)* mutant, we can conclude that the short isoform (and possibly medium) also contributes to CLA-1 function at the NMJ.

### Loss of Release in the *cla-1;unc-10* Double Mutants Is Not due to Additive Docking Defects.

**Fig. 2.**
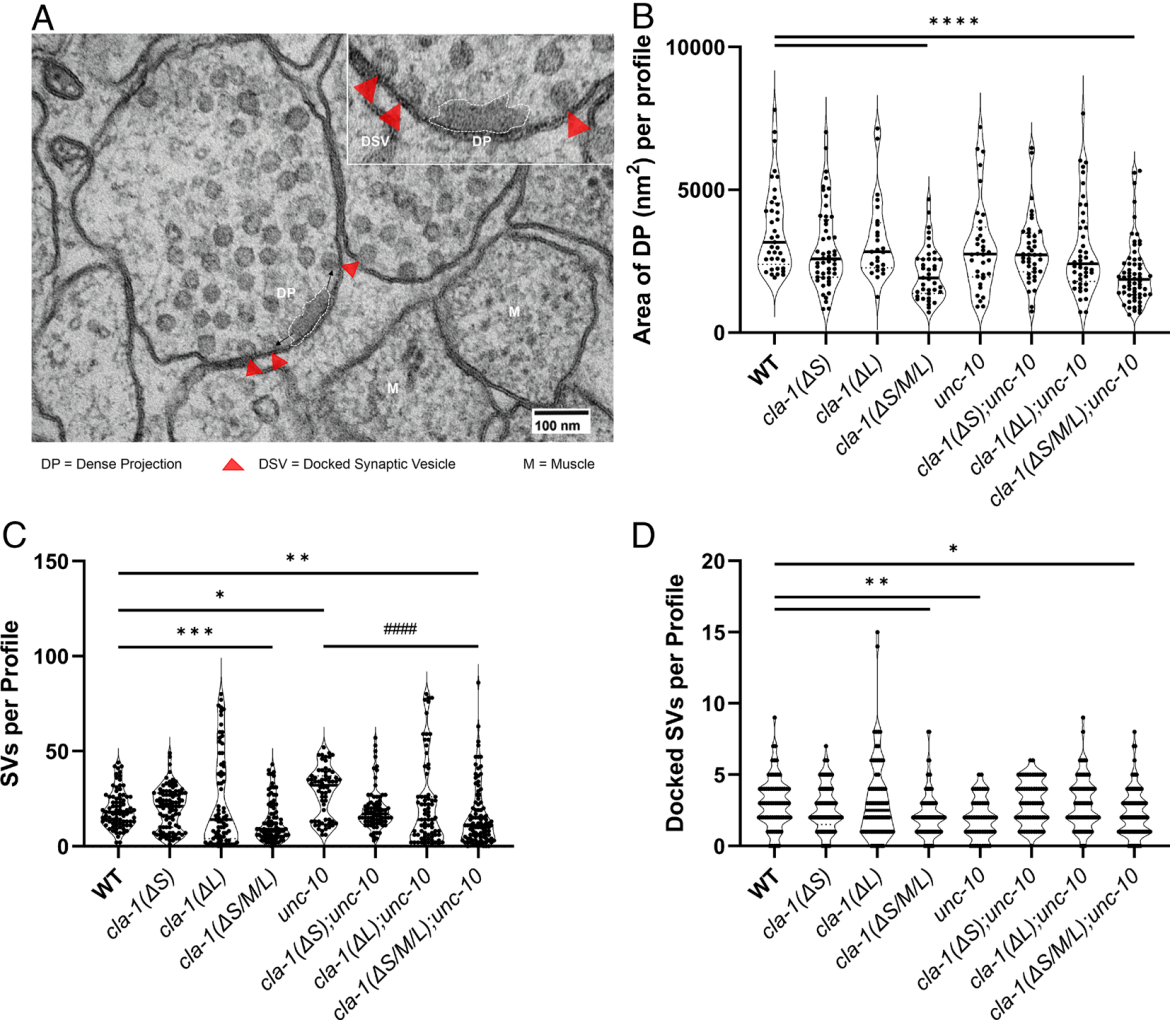
Analysis of *cla-1* isoform single and double *unc-10* mutants using HPF/FS electron microscopy demonstrates that loss of all CLA-1 isoforms is necessary to disrupt synaptic morphology. (*A*) A single 40-nm cross-section of a synapse from a young adult worm prepared by HPF/FS. Examples of a dense projection and docked SVs (SVs 0 nm distance to the plasma membrane) measured and quantified in this study. (*B*–*D*) These data show that loss of CLA-1(L) or (S) alone does not result in the same morphological defects as observed in the *cla-1(ΔS/M/L)* mutant, indicating that loss of all CLA-1 protein is necessary to disrupt the (*B*) dense projection and reduce the number of (*C*) cytosolic and (*D*) docked SVs. *P < 0.05, **P < 0.01, ***P <0.001, ****P < 0.0001 (*C*) SV number in *cla-1(ΔS/M/L);unc-10* are significantly reduced when compared to *unc-10* (^####^*P* < 0.0001) and not different from *cla-1(ΔS/M/L).* One way ANOVA with Tukey’s post hoc analysis.

Given our previously published electron microscopy (EM) data showing that *cla-1* null mutants have reduced DP size and fewer SVs (7), we hypothesized that the severity of the *cla-1(ΔS/M/L);unc-10* double mutants could be due to additive effects of loss of CLA-1 and UNC-10 on synapse morphology. To test this, we compared the NMJ ultrastructure of the *cla-1* mutants with and without UNC-10. We focused on the ventral NMJs from which the synaptic recordings were obtained. Worms were prepared using high-pressure freeze (HPF) fixation, a method that preserves synapse morphology in its near native state (8, 9). Consistent with previous EM data (7), the area of the DP (Fig. 2*A*) in *cla-1(ΔS/M/L)* mutants was significantly smaller than wild type, possibly indicative of reduced AZ protein content (Fig. 2*B*). However, neither *cla-1(ΔL)* nor *cla-1(ΔS)* mutants affected DP area, indicating that this phenotype requires loss of multiple isoforms (Fig. 2*B*). Consistent with previous studies (5), the DP area of *unc-10* mutants was not significantly altered (Fig. 2*B*). Importantly, the DP size of the *cla-1(ΔS/M/L);unc-10* double mutants was no more reduced than *cla-1(ΔS/M/L)* alone, and neither *cla-1(ΔS)* nor *cla-1(ΔL)* mutants impacted the DP area in the *unc-10* mutant background (Fig. 2*B*). Therefore, the severe synaptic phenotype of the *cla-1(ΔS/M/L);unc-10* double mutants cannot be attributed to additive effects on DP morphology. Moreover, this result indicates that the major role CLA-1 plays in determining DP content, requires both long and short isoforms, suggesting that the common CLA-1 C-terminal localized at the AZ is responsible for this function.

We previously demonstrated that *cla-1(ΔS/M/L)* mutants have fewer total SVs at NMJs (7). When we examined the isoform specificity of this phenotype, we found that only *cla-1(ΔS/M/L)* mutants exhibited a reduction in SVs (Fig. 2*C*). Could the severity of the *cla-1;unc-10* double-mutant release defect be due to further reductions in SV numbers? As previously observed, *unc-10* single mutants exhibit a significant accumulation of SVs proposed to result from impaired release (Fig. 2*C*) (10, 11). However, in the *cla-1(ΔS/M/L);unc-10* double mutants, SV numbers were reduced compared to *unc-10* and similar to *cla-1(ΔS/M/L)*. The data suggest that CLA-1 isoforms impact SV density through a mechanism independent of UNC-10 (Fig. 2*C*). Therefore, the severe loss of release in *cla-1;unc-10* double mutants cannot be attributed to a sparsity of SVs.

In previous studies, we have shown that *unc-10* mutants have fewer morphologically docked SVs proximal to the DP (10, 11). *cla-1(ΔS/M/L)* mutants on the other hand have fewer total docked SVs, but the proximally docked SV pool is abundant (7). Neither the *cla-1(ΔL)* nor *(ΔS)* mutants exhibited a change in total number or distribution of docked SVs as compared to the wild type (Fig. 2*D* and *SI Appendix*, Fig. S2). We next asked whether the loss of CLA-1 isoforms and UNC-10 together further impacts docking. We found that the docked SV pool deficits of the *cla-1* and *unc-10* mutants were not exacerbated in the double mutants. In fact, in all three double mutants, SV docking trended higher than *unc-10* alone (Fig. 2*D*). Together these data indicate that *cla-1(ΔS/M/L)*;*unc-10* double mutants incorporate changes seen in each single mutant but show no additivity, suggesting that CLA-1 and UNC-10 differentially regulate these aspects of the synaptic ultrastructure. Importantly, the dramatic release defects in the *cla-1(ΔL) and cla-1(ΔS/M/L)* double mutants are not attributable to further loss of docked SVs in the *unc-10* mutant background.

Docked SVs at *C. elegans* synapses consist of a small fraction of unprimed docked SVs that are targeted proximal to the DP through UNC-10 interactions with RAB-3, as well as a larger, and more distributed, pool of docked SVs that have undergone UNC-13-dependent priming (10, 12). Given these findings, we next examined whether the relatively normal morphologically docked SV pool in the *cla-1*;*unc-10* mutants is fusion competent. Previous studies have demonstrated that application of hypertonic saline results in the release of fusion-competent SVs (13). Therefore, to address whether the elimination of release in the *cla-1*;*unc-10* double mutants is due to loss of priming downstream of docking, we examined the hyperosmotic responses in the *unc-10* single mutants as well as *cla-1(ΔS/M/L);unc-10* and *cla-1(ΔL)*;*unc-10* double mutants at the NMJ. We found that both double mutants exhibited robust hyperosmotic responses, indicating that the morphologically docked SVs observed are primed and fusion-competent and therefore, cannot account for the total loss of evoked release in the double mutants (S3).

### CLA-1 Regulates UNC-2/Ca_V_2 Presynaptic Localization Together with UNC-10/RIM.

**Fig. 3.**
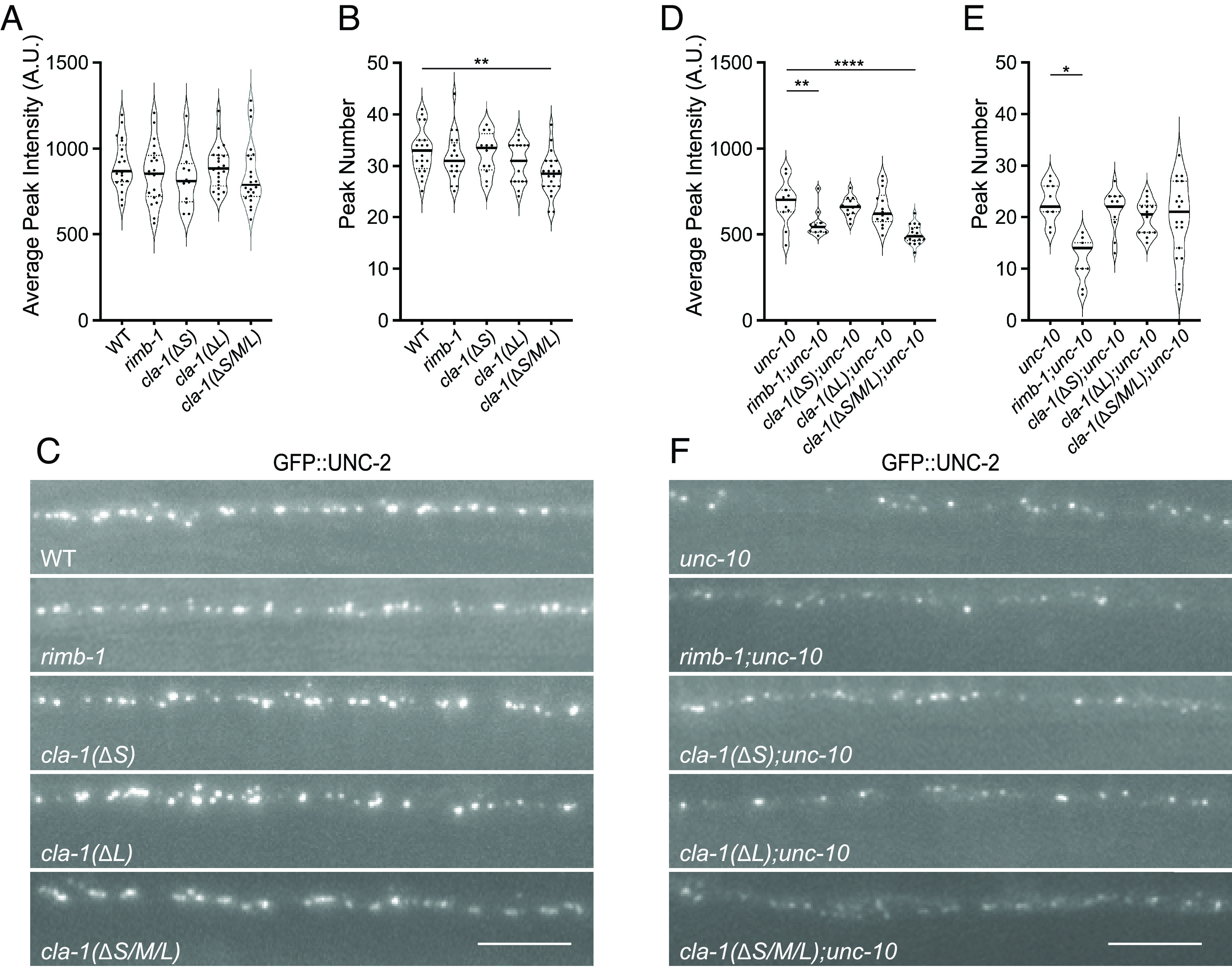
Endogenously tagged GFP::UNC-2 levels are additively reduced in a *cla-1(ΔS/M/L);unc-10* double mutant. (*A* and *B*) GFP::UNC-2 average peak intensities (*A*) and peak numbers (*B*) were determined based on analysis of a 30-μm section of the dorsal nerve cord (DNC) of wild-type, *rimb-1(ce828)cla-1(ΔS)*, *cla-1(ΔL),* and *cla-1(ΔS/M/L)* single-mutant animals. The solid and dotted lines in the violin plot indicate the median and quantile values, respectively. Individual dots represent biological replicates from single animals. ns, not significant, **P < 0.01, One-way ANOVA, Tukey’s post hoc analysis. (*C*) Representative DNC images from wild-type and single-mutant animals. (Scale bar, 5 μm.) (*D* and *E*) GFP::UNC-2 average peak intensities (*D*) and peak numbers (*E*) were determined based on analysis of a 30-μm section of the dorsal nerve cord (DNC) of wild-type, *unc-10(md1117)rimb-1*;*unc-10, cla-1(ΔS);unc-10*, *cla-1(ΔL);unc-10, and cla-1(ΔS/M/L); unc-10* double-mutant animals. *P < 0.05, ****P < 0.0001, One-way ANOVA, Tukey’s post hoc analysis. (*F*) Representative DNC images from the indicated mutants. (Scale bar, 5 μm.)

As *cla-1* mutants do not exacerbate the *unc-10* SV docking phenotype, we hypothesized that the loss of release could be due to defects in calcium-secretion coupling in these mutants. In *C. elegans,* Ca^2+^ influx at the presynaptic terminal is largely mediated by the UNC-2 channel, the sole Ca_V_2 channel a1 homolog in *C. elegans* (10, 14–16). Immuno-EM and fluorescent imaging data have shown that UNC-2 is enriched at the DP and colocalizes with UNC-10 (10, 16, 17). We recently demonstrated endogenously tagged UNC-2 peak number and average peak intensity are significantly reduced in *unc-10* mutants by ~30% (Fig. 3 *A*–*C*) (17). Here, we used endogenous GFP-tagged UNC-2 to determine whether loss of CLA-1 also regulates UNC-2 levels. We did not observe obvious changes in the *cla-1* mutants alone, with the exception of *cla-1(ΔS/M/L)*, which exhibited a small reduction in UNC-2 peak number (wild type vs. *cla-1*: 32.8 ± 0.94 vs. 28.32 ± 0.8, mean ± SEM, *P* < 0.0047) suggesting both CLA-1 isoforms contribute to localization of UNC-2 (Fig. 3 *A*–*C*). To address the possibility that the *cla-1(ΔS/M/L)* result was due to a background mutation in the *cla-1(wy1048)* allele, we examined a second *cla-1(ΔS/M/L)* mutant, (*cla-1(ok2285)*, and demonstrated a similar reduction in UNC-2 levels (S4). In the *cla-1(ΔS/M/L);unc-10* double mutants we saw a greater reduction in UNC-2 peak intensity compared to *unc-10* single mutants, but no change in either *cla-1(ΔL);unc-10* or *cla-1(ΔS);unc-10* double mutants compared to *unc-10* (Fig. 3 *D*–*F*). These data suggest that CLA-1 works together with UNC-10 to regulate synaptic UNC-2 levels.

### Both CLA-1 and UNC-10 Regulate the Synaptic Localization of RIMB-1.

**Fig. 4.**
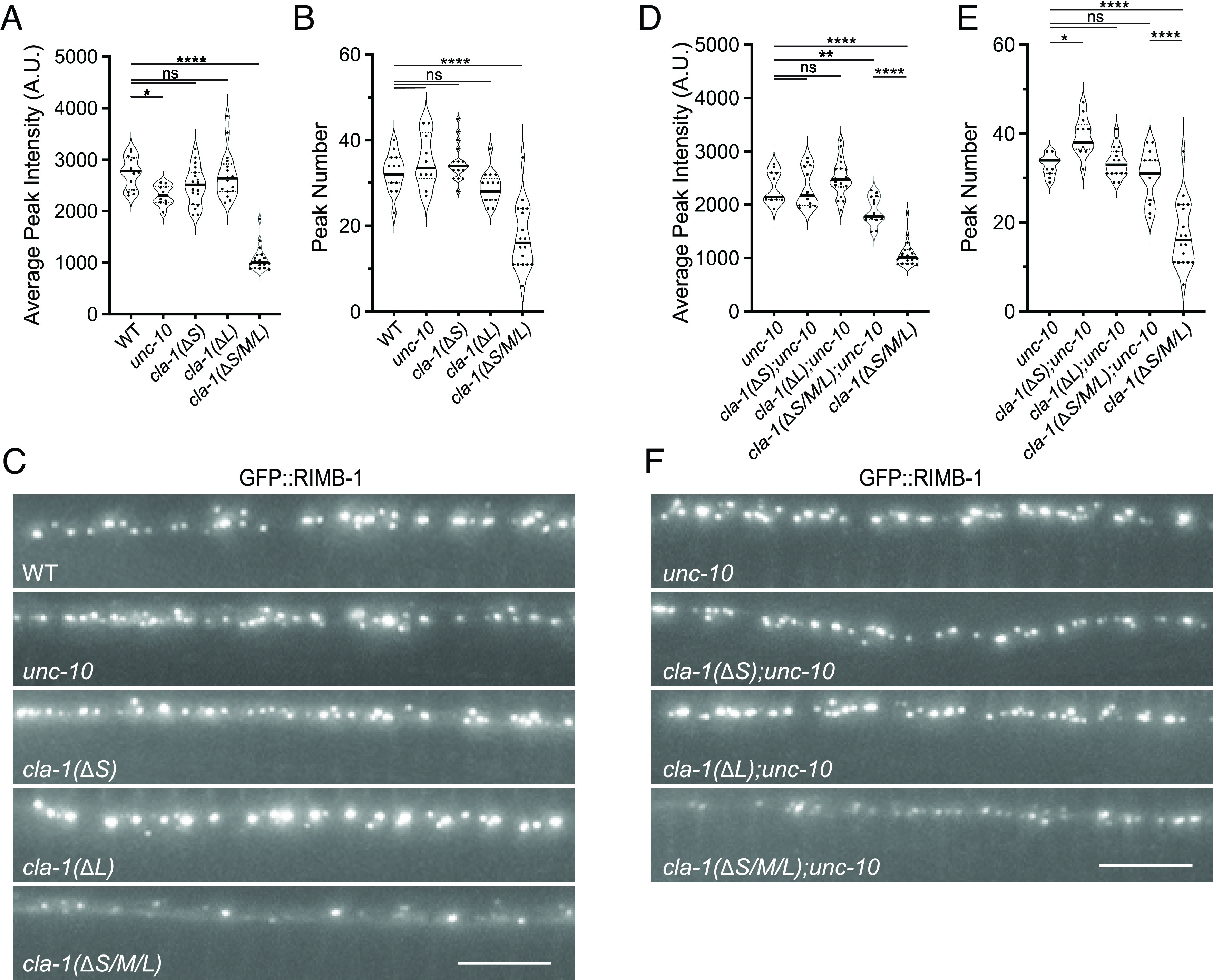
Endogenous RIMB-1 levels are drastically reduced in *cla-1(ΔS/M/L)* mutants. (*A* and *B*) GFP::RIMB-1 average peak intensities (*A*) and peak numbers (*B*) were determined based on analysis of a 30-μm section of the DNC of wild-type, *unc-10(md1117)cla-1(ΔS)*, *cla-1(ΔL),* and *cla-1(ΔS/M/L)* single-mutant animals. RIMB-1 peak number and intensity are dramatically reduced in *cla-1(ΔS/M/L)* mutants (****P < 0.0001), more so than *unc-10* mutants. (*C*) Representative GFP::RIMB-1 images from wild-type, *unc-10,* and *cla-1* mutant animals. (Scale bar, 5 μm.) (*D* and *E*) GFP::RIMB-1 average peak intensities (*D*) and peak numbers (*E*) in *unc-10* and *cla-1* double mutants. *P < 0.05, **P < 0.01, ****P < 0.0001, One-way ANOVA, Tukey’s post-hoc analysis. (*F*) Representative GFP::RIMB-1 images from *unc-10* and *cla-1* double mutants. (Scale bar, 5 μm.)

How might CLA-1 impact UNC-2 in the absence of UNC-10? We previously demonstrated that *rimb-1* null mutants also exacerbate the *unc-10* mutant deficit in UNC-2 levels suggesting these two AZ proteins act redundantly to regulate UNC-2 (17). Similar redundant effects were observed for vertebrate RIM and RIM-BPs (18). Therefore, we compared UNC-2 levels in *rimb-1;unc-10* to those of *cla-1(ΔS/M/L);unc-10* double mutants. While peak number was more severely reduced in *rimb-1;unc-10* mutants, we found that UNC-2 peak intensity in *rimb-1;unc-10* mutants had a similar reduction to *cla-1(ΔS/M/L);unc-10* mutants (Fig. 3 *D*–*F*). Based on these results, we hypothesized that CLA-1 may be regulating UNC-2 via RIMB-1. To test this possibility, we assessed presynaptic RIMB-1 levels in the *cla-1* mutants using endogenously GFP-tagged RIMB-1. In *cla-1(ΔS)* and *cla-1(ΔL)* RIMB-1 peak intensity and number were comparable to the wild type. However, we found a severe reduction in both peak number and average intensity in the *cla-1(ΔS/M/L)* mutants (Fig. 4 *A*–*C*). Here, we verified the veracity of this result by examining RIMB-1 levels in *cla-1(ok2285)*, as well as a second RIMB-1 fluorescent probe in *cla-1(wy1048)*, both of which yielded similar results (S5). These data indicate that CLA-1 plays a major role in the localization of RIMB-1 at the synapse.

The reduction of RIMB-1 levels in *cla-1(S/M/L)* mutants resembles that observed in the absence of the synaptic scaffolding protein SYD-2, suggesting that loss of SYD-2 may contribute to the RIMB-1 phenotype of *cla-1(S/M/L)* mutants (17). In fact, we have previously shown that *cla-1(S/M/L)* mutants have a minor reduction (~20%) in synaptic levels of SYD-2 (7). This raises the possibility that the functional changes in *cla-1* mutants could be the result of defects in SYD-2-dependent synaptogenesis and/or synapse stability. Therefore, we examined the synaptic levels of another key AZ protein, ELKS-1 in *cla-1* mutants using endogenously GFP-tagged ELKS-1. The peak intensity and number of ELKS-1 puncta were unaffected in *cla-1(ΔL)* and *cla-1(ΔS/M/L)* mutant animals when compared to wild-type animals (S6). Together, these data demonstrate that CLA-1 is required for RIMB-1 localization at the AZs without dramatically impacting synaptic density.

Next, we examined whether UNC-10 and CLA-1 independently regulate presynaptic RIMB-1 localization. As previously reported, RIMB-1 intensity, but not peak number, was considerably reduced in *unc-10* mutants (17). Introduction of the *unc-10* mutation did not cause any appreciable changes in RIMB-1 peak intensities and numbers in either *cla-1(ΔL)* or *cla-1(ΔS)* mutant animals (Fig. 4 *D*–*F*). But compared to *unc-10* single mutants, *cla-1(ΔS/M/L);unc-10* double mutants showed a further reduction in RIMB-1 puncta intensity with no change in number (Fig. 4 *D*–*F*). Interestingly, RIMB-1 puncta intensity in *cla-1(ΔS/M/L);unc-10* double mutants is less reduced than *cla-1(ΔS/M/L)* alone, implying that in the absence of CLA-1 and UNC-10, RIMB-1 is localized to the AZ by an alternate interaction. We have shown that both SYD-2 and ELKS-1 are involved in the localization of RIMB-1 (17), therefore it is possible that in the *cla-1(ΔS/M/L);unc-10* double mutants either of these or other AZ proteins could partially compensate.

Though SYD-2 is reduced in *cla-1(S/M/L)* mutants, this cannot explain the severity of the evoked release defects observed in the *cla-1;unc-10* double, as we have previously shown that evoked release in *syd-2*;*unc-10* double mutants is no more severe than *unc-10* mutants alone (19). Furthermore, whereas electrophysiological analysis of a *cla-1(S/M/L);syd-2* double mutant exhibited evoked amplitudes similar to those of *syd-2*, the mini frequency of these double mutants was reduced beyond that of *syd-2* suggesting additional roles for CLA-1 in synaptic release (S7).

### CLA-1 Promotes Release Independent of RIMB-1 and UNC-2 Regulation.

**Fig. 5.**
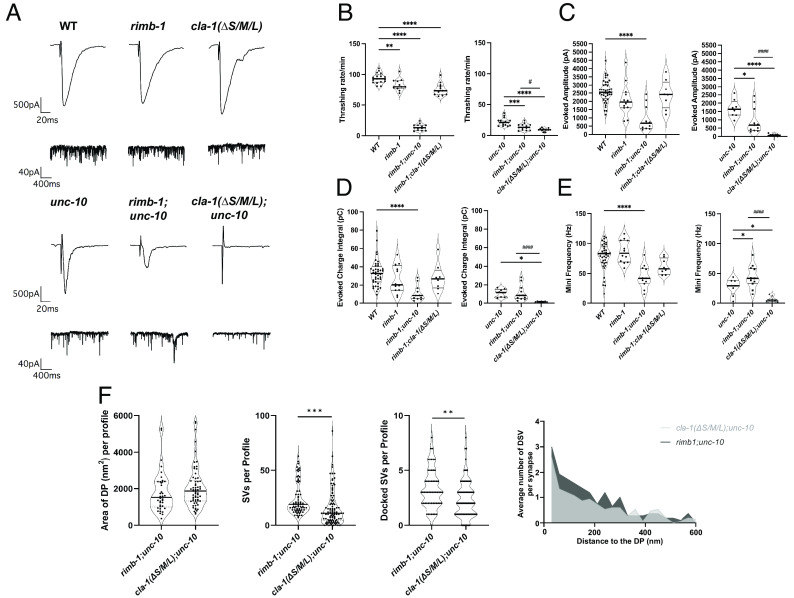
Thrashing rates, electrophysiological recordings, and ultrastructural analysis in *cla-1;unc-10* and *rimb-1;unc-10* doubles demonstrate that *cla-1* mutants have a more severe synaptic defect than *rimb-1* mutants. (*A*–*E*) These data show that while *rimb-1;unc-10* exhibits more severe behavioral and release defects than *unc-10* mutants alone, there is still a significant difference in the severity of release in a *cla-1;unc-10* double. *P < 0.05, **P < 0.01, ***P < 0.001, ****P < 0.0001. (*F*) Ultrastructural analysis shows no significant difference in the area of DP between the two double mutants. However, we observed that *rimb-1;unc-10* double-mutant synapses have more cytosolic and docked SVs. Additionally, while docking distributions are similar in both double mutants, the docked SVs in *rimb-1;unc-10* mutants appear to accumulate proximal to the active zone. One way ANOVA, Tukey’s post hoc analysis.

If the role of CLA-1 is primarily to regulate RIMB-1 levels, we would expect *rimb-1;unc-10* double mutants to phenocopy *cla-1(ΔS/M/L);unc-10* double mutants. To address this possibility, we compared the phenotypes of the two double mutants. We first performed a behavioral assay looking at thrashing rates. Although *cla-1(ΔS/M/L)* and *rimb-1(ce828)* single mutants exhibited mild but significant reductions in thrashing rates compared to the wild type, they were not different from each other and were less severe than the *unc-10* mutants (Fig. 5*B*). While both *cla-1(ΔS/M/L);unc-10* and *rimb-1;unc-10* double mutants were more impaired than *unc-10*, the *cla-1(ΔS/M/L);unc-10* thrashing defect was significantly worse than *rimb-1;unc-10* (Fig. 5*B*).

To determine whether the above behavioral differences were recapitulated at the level of the NMJ, we recorded evoked and endogenous release in these mutants. Whereas the evoked amplitude in *cla-1* mutants was similar to the wild type, the charge integral, a reflection of the total number of fused SVs, was significantly reduced, as was the endogenous mini rate (refer Fig. 1 *C* and *G*) (7). In contrast *rimb-1* mutants had robust evoked release amplitudes, evoked charge integrals and endogenous mini frequencies (Fig. 5 *A* and *C*–*E*). These data demonstrate that only *cla-1* null mutants have detectable release defects at the NMJ. However, when we examined *rimb-1* in the *unc-10* mutant background, we uncovered additive synaptic release defects. Specifically, in *rimb-1;unc-10* double mutants the evoked release amplitude was reduced to 36% of WT and 56% of *unc-10*, with no further reduction in charge integral or mini frequency compared to *unc-10* (Fig. 5 *A* and *C*–*E*). These combined effects on evoked release are in keeping with previously documented roles for both Rim and RIM-BPs in Ca^2+^ channel localization and Ca^2+^-release coupling. Although these data clearly show *rimb-1;unc-10* double mutants severely impact synaptic function, the extent of the effect is far less than the near total elimination of evoked release observed in *cla-1(ΔS/M/L);unc-10* double mutants (3% of WT and 5% of *unc-10* evoked amplitude) and mini frequency (9% of WT and 24% of *unc-10*). Consistent with these clear functional differences, when we compared the ultrastructure of *cla-1;unc-10* and *rimb-1;unc-10* double mutants, we also saw significantly fewer SVs and docked SVs in *cla-1;unc-10* when compared to *rimb-1;unc-10,* though the distribution of these docked SVs is similar in the double mutants and much like that observed in *rimb-1* and *unc-10* alone (Fig. 5*F* and *SI Appendix*, Fig. S8). Given that these ultrastructural and physiological results mirror the differential severity of the behavior in the intact worms, these data support the interpretation that CLA-1 has roles in neurotransmission beyond the observed regulation of RIMB-1 and UNC-2 levels.

### Presynaptic UNC-13 Localization Requires Independent Actions of CLA-1 and UNC-10.

**Fig. 6.**
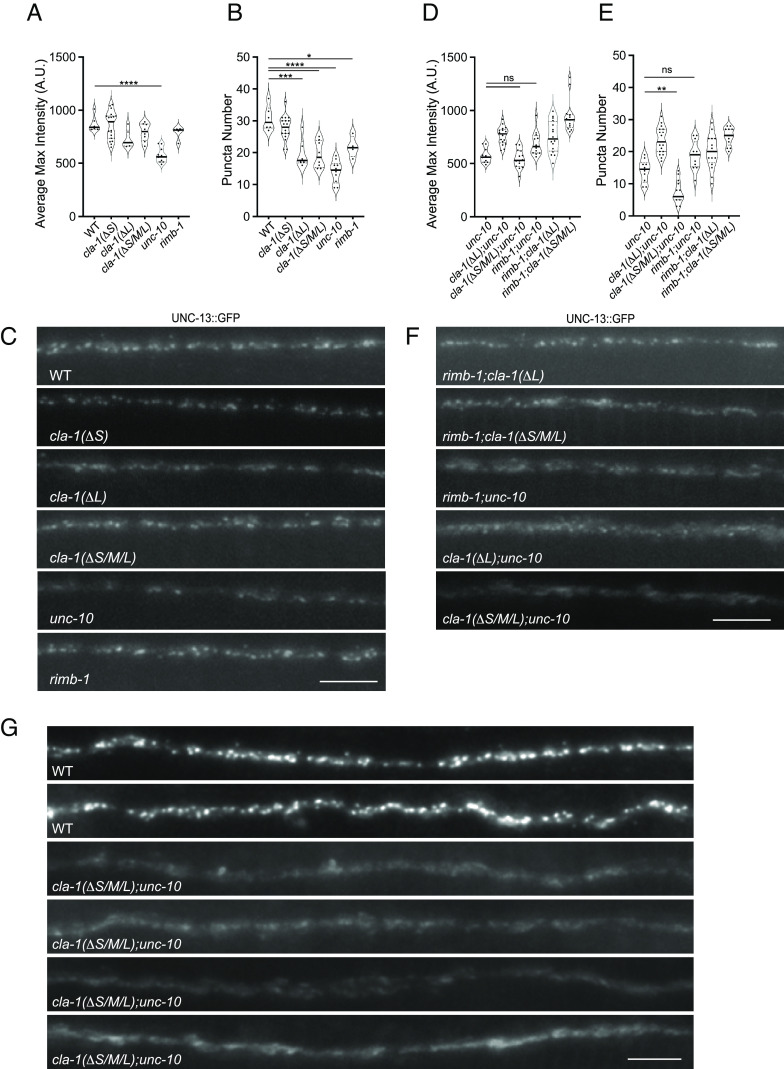
UNC-13 is reduced in *cla-1* and *unc-10* single mutants and shows a further reduction in *cla-1;unc-10* double mutants. (*A* and *B*) UNC-13::GFP average puncta max intensities (*A*) and puncta numbers (*B*) were determined based on analysis of a 30 μm section of the DNC of the indicated wild-type and single-mutant animals. While UNC-13 puncta intensity and number are reduced in *unc-10* animals, the puncta numbers are significantly reduced in *cla-1(ΔL)* and *cla-1(ΔS/M/L)* mutants. *P < 0.05, ***P < 0.001, ****P < 0.0001, One-way ANOVA, Tukey’s post hoc analysis. (*C*) Representative UNC-13::GFP images from the indicated wild-type and single-mutant animals. (Scale bar, 5 μm.) (*D* and *E*) UNC-13::GFP average puncta max intensities (*D*) and puncta numbers (*E*) were determined based on analysis of a 30-μm section of the DNC of the indicated *unc-10* and double-mutant animals. **P < 0.01, One-way ANOVA, Tukey’s post hoc analysis. (*F*) Representative GFP::RIMB-1 images from *unc-10* and *cla-1* double mutants. (Scale bar, 5 μm.) (*G*) Examples of straightened DNC images (60 μm) in *cla-1(ΔS/M/L);unc-10* double-mutant animals in comparison to wild-type animals.

**Fig. 7.**
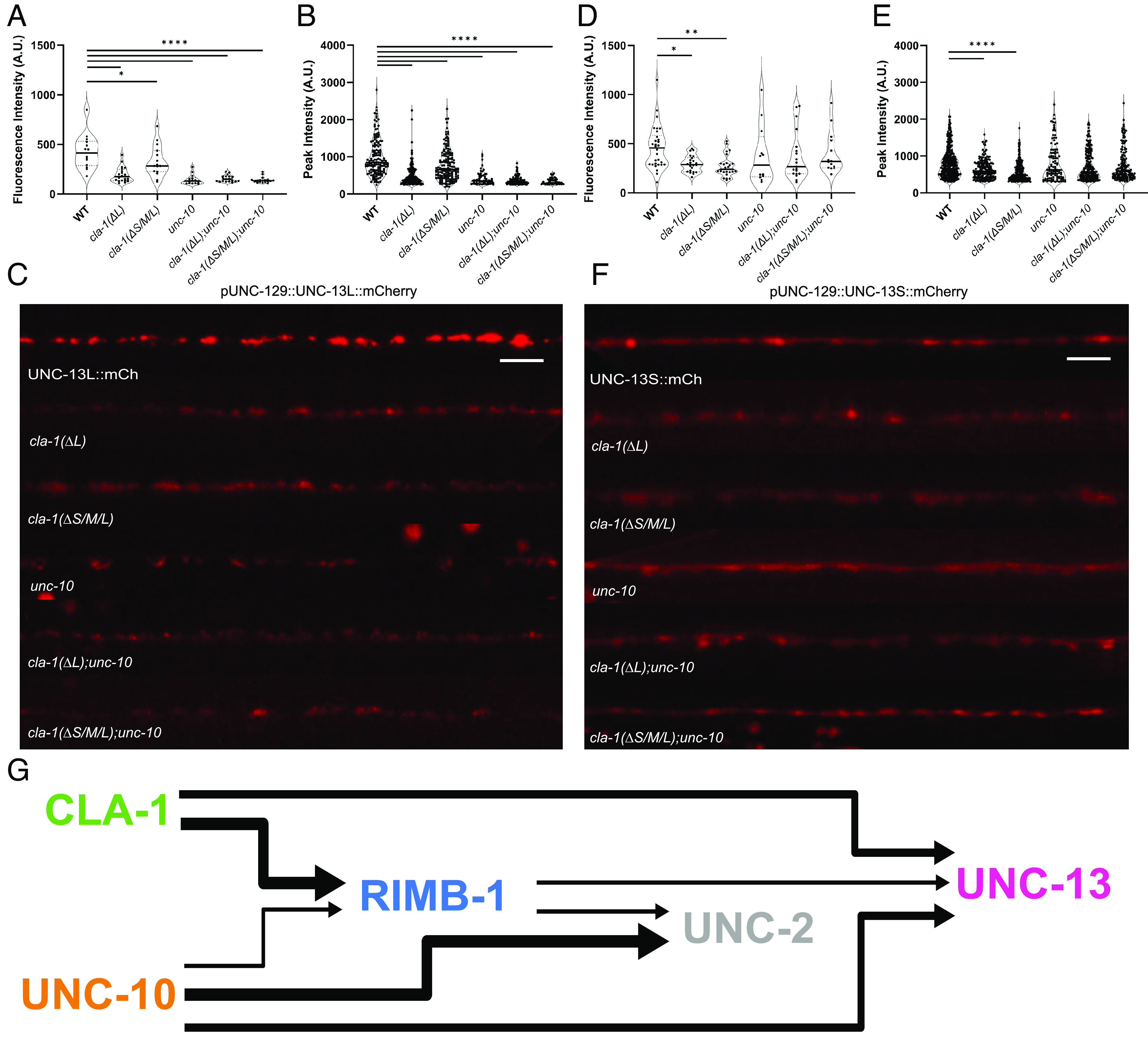
UNC-13L and S isoforms are reduced in *cla-1* mutants. (*A*–*C*) The fluorescence and peak intensities of the UNC-13L isoform are significantly reduced in *cla-1(ΔL)cla-1(ΔS/M/L)unc-10* and both double mutants. *P < 0.05, ****P < 0.0001, One-way ANOVA, Dunnett’s post hoc analysis. (*D*–*F*) The fluorescence and peak intensities of the UNC-13S isoform are only reduced in the *cla-1(ΔL)* and *cla-1(ΔS/M/L)*. *P < 0.05, **P < 0.01, ****P < 0.0001, One-way ANOVA, Dunnett’s post hoc analysis. (Scale bar, 10 µm.) Extraneous fluorescent puncta outside the nerve cord represent gut granule autofluorescence and were not included in the analysis. (*G*) A genetic model for CLA-1- and UNC-10-dependent regulation of downstream AZ components.

What additional role could CLA-1 have in release? In *C. elegans*, the evoked synaptic phenotype of *unc-13* null mutants is remarkably similar to that of *cla-1;unc-*10 double mutants (3). This led us to ask whether CLA-1 regulates UNC-13 function. To address this, we generated an endogenously C-terminal tagged UNC-13 strain, labeling both UNC-13L and UNC-13S isoforms. Compared to wild-type animals, both the intensity and number of UNC-13::GFP puncta were substantially reduced in *unc-10* mutants (~30% and ~40%, respectively). *cla-1(ΔL)* and *cla-1(ΔS/M/L)* mutants showed a similar reduction in UNC-13 puncta number without changing puncta intensity (Fig. 6 *A*–*C*). To determine whether CLA-1 and UNC-10 independently regulate UNC-13, we examined endogenous UNC-13 in *cla-1;unc-10* double mutants. Strikingly, in *cla-1(ΔS/M/L);unc-10*, UNC-13 puncta were barely discernible (Fig. 6 *D*–*G*). This result likely represents loss of the UNC-13L isoform as it is known to form discrete synaptic puncta, whereas UNC-13S is diffuse (Fig. 6 *F*–*G*) (20). To support this interpretation we examined transgenic lines expressing either mCherry-tagged UNC-13L or UNC-13S in *cla-1(ΔL)*, *cla-1(ΔS/M/L)* and *unc-10* single and double mutants. Consistent with the endogenous data, both *cla-1* and *unc-10* single and double mutants reduced UNC-13L levels (Fig. 7 *A*–*C*). UNC-13S fluorescence levels were only significantly reduced in *cla-1(ΔL)* and *cla-1(ΔS/M/L)* single mutants but *cla-1;unc-10* double mutants had wild-type levels (Fig. 7 *D*–*F*). The specific loss of UNC-13L in *cla-1;unc-10* double mutants resembles the loss of synaptic transmission previously observed in *unc-13(e1091)* mutants, in which only UNC-13S remains (3, 20).

Given that CLA-1 and, to a lesser extent UNC-10 regulate RIMB-1, we next examined endogenous UNC-13 labelling in *rimb-1* mutants. In *rimb-1* single mutants the puncta intensity was not significantly affected, whereas the puncta number showed a ~30% reduction, possibly reflecting effects of RIMB-1 on a subset of synapses (Fig. 6 *A*–*C*). In *rimb-1;unc-10* double mutants we observed no further reduction in UNC-13 levels relative to *unc-10* (Fig. 6 *D*–*F*). In *rimb-1;cla-1(ΔS/M/L)* double mutants we did not observe appreciable changes in UNC-13 levels. Thus, the additive effect on UNC-13 levels in *cla-1(ΔS/M/L);unc-10* double mutants supports a role for CLA-1 in the regulation of UNC-13 that is independent of both RIMB-1 and UNC-10. Surprisingly, when we examined *cla-1(ΔL);unc-10* double mutants, we found that the endogenous UNC-13 levels were not further reduced relative to *unc-10*, suggesting that in *cla-1(ΔL);unc-10* mutants, RIMB-1 and the remaining CLA-1 isoforms are able to support UNC-13 synaptic expression in the absence of UNC-10. Together these data suggest that UNC-10, RIMB-1 and CLA-1 exhibit partial redundancy in regulating UNC-13 localization.

These data provide compelling evidence for several genetic interactions through which CLA-1 contributes to synaptic function, predominantly through the regulation of RIMB-1 and UNC-13, and along with UNC-10 governs neurotransmitter release at *C. elegans* synapses (Fig. 7*G*).

## Discussion

### CLA-1 Regulates UNC-2/Ca_V_2 Presynaptic Localization Together with UNC-10/RIM by Recruiting RIMB-1.

Here, we show that *C. elegans* CLA-1 and UNC-10 act together to localize UNC-2. While *cla-1(ΔS/M/L)* mutant animals exhibit a negligible effect on UNC-2 localization relative to *unc-10* mutants, the *cla-1(ΔS/M/L)*;*unc-10* double mutants exacerbate the UNC-2. The additivity of the UNC-2 localization defect in the *cla-1(ΔS/M/L)*;*unc-10* doubles likely results from the loss of RIMB-1, which we have shown is heavily dependent on CLA-1. Mammalian Bassoon interacts with RIM-BPs via a proline-rich (PxxP) SH3-binding motif (21), which appears to be conserved in Piccolo and Fife (22, 23) Interestingly, CLA-1 also has several putative PxxP SH3-binding motifs in its C terminus, which we speculate serve to recruit RIMB-1 to the release site.

In other systems, RIM and RIM-BP have conserved roles in the recruitment of calcium channels (21, 23–27). RIM proteins localize Ca^2+^ channels through a PDZ domain, whereas the SH3 domains of RBPs are known to bind calcium channels at PxxP motifs (23, 27, 28). In *C. elegans*, while loss of RIMB-1 alone has no impact on UNC-2 levels, UNC-2 localization is substantially reduced in the absence of UNC-10 and this disruption is further exacerbated in the absence of RIMB-1 (17, 26). These data suggest that UNC-10 is a major player in UNC-2 localization, while RIMB-1 plays a minor role. Similar observations have been reported in vertebrates, suggesting this is a conserved arrangement (18, 29, 30). Indeed, in vertebrates, RIM-BPs are not essential for neurotransmitter release but are selectively required for tight coupling of SVs to presynaptic Ca_V_2 channels (31).

### CLA-1 Plays a Key Role in Determining the AZ Composition.

Prior research has established a correlation between DP size and synaptic strength at the *C. elegans* NMJ, a finding that mirrors the scaling of AZ size with the readily releasable pool in other systems (1, 32–35). Specifically, *syd-2* mutants, with an early stop loss-of-function mutation, have reduced DP size associated with greater synaptic fatigue, whereas a hypermorphic *syd-2* gain-of-function mutation enhances the size and complexity of the DP, producing sustained release during train stimulation (6, 19). Similarly, *cla-1* mutants have smaller DPs and fatigue faster than WT. In contrast, the DP of *unc-10* mutants is not significantly altered, nor do we see further reductions in the *cla-1;unc-10* double mutant. These data indicate that CLA-1 regulates the DP independently of UNC-10/RIM. In this respect *cla-1* resembles the changes observed in *Drosophila Fife* mutants, which have smaller DPs and similar to our results, DP size in *Drosophila RIM* mutants is unaffected (27, 36).

How might CLA-1 regulate DP size? We previously documented a reduction in the expression of the *C. elegans* Liprin- α ortholog, SYD-2, in *cla-1(ΔS/M/L)* mutants (7). SYD-2 has been shown to be an integral component of the DP (37). Therefore, we would expect that loss of SYD-2 in *cla-1(ΔS/M/L)* mutants would contribute to the reduction in DP size. Here, we found that *cla-1(ΔS/M/L)* mutants also exhibit a dramatic reduction in RIMB-1 levels. Using endogenously tagged proteins, we know from a previous study that RIMB-1 is more abundant than other key AZ components examined (RIMB-1 > ELKS, SYD-2 > UNC-10 and UNC-2) (17). Thus, we predicted and demonstrated that *rimb-1* mutants have a significant reduction in DP area. Bruckner et al., have reported a similar reduction in DP size in *Drosophila Rimb1* mutants. In contrast, RIM-BP2 deletion in hippocampal neurons does not alter DP size, but when combined with loss of RIM exhibits a striking reduction in DP number and area (18). Based on these observations, it appears that CLA-1 stabilization of SYD-2 and RIMB-1 likely contributes to the DP protein density. This does not exclude the possibility that other cytomatrix components impacted in *cla-1* mutants also contribute to the reduced DP size, including CLA-1 itself.

### CLA-1 Regulates SV Docking Independent of RIMB-1.

At *C. elegans* NMJs the morphologically docked SV pool is partially dependent on interactions between SV-associated RAB-3 and UNC-10/RIM (10, 11). In *unc-10* and *rab-3* single mutants, docked SVs are specifically reduced proximal to the DP (<100 nm), whereas *cla-1(ΔS/M/L)* mutants retain this proximal pool but show reductions in overall docking. We show here that, like *unc-10* mutants, *rimb-1* mutants exhibit a reduction in proximally docked SVs, suggesting that RIMB-1 also has a role in positional docking in *C. elegans*. Furthermore, since the *rimb-1;unc-10* double mutants do not show additivity in proximal docking defects, their roles appear to be redundant. This would explain why in a *cla-1(ΔS/M/L)* mutant in which RIMB-1 levels are greatly reduced, proximal docking can still occur due to the continued presence of UNC-10. This interpretation is supported by the observation that in *cla-1;unc-10* double mutants, the proximal SV pool is reduced to the same extent as *rimb-1* and *unc-10* single mutants as well as the *rimb-1;unc-10* double mutants.

The loss of proximal docking in *cla-1;unc-10* and *rimb-1;unc-10* double mutants may contribute to the release defects observed in these strains. Indeed, redundant roles for RIMB-1 and RIM in synaptic SV docking appear to be well-conserved as knockouts of RIMs and RIM-BPs in mice exhibit severe reductions in SV docking and priming associated with a dramatic reduction of neurotransmitter release (18).

### CLA-1 Acts In Concert with UNC-10 and RIMB-1 to Organize Key Components of the Release Machinery.

While UNC-10 interactions with RAB-3 can morphologically dock SVs proximal to the DP, UNC-13 is required to promote SNARE complex assembly, resulting in fusion competent docked SVs (3, 38, 39). UNC-10 is known to directly interact with UNC-13 (40). In this study, we have demonstrated that both UNC-10 and CLA-1 regulate endogenous UNC-13 synaptic localization. Importantly, this loss of UNC-13 puncta does not appear to be due to fewer synapses, based on our ELKS data, and therefore likely reflects UNC-13 levels that fall below peak threshold at existing synapses (17).

How might CLA-1 regulate UNC-13? At hippocampal mossy fiber synapses, Munc13-1 is recruited to the AZ by RIM-BP2 (41) and in *Drosophila* RIM-BP has been shown to directly bind an Unc13 PxxP motif via a C-terminal SH3 domain (23). Since we know *cla-1(ΔS/M/L)* mutants have reduced RIMB-1 levels, this presents a potential mechanism by which CLA-1 could affect UNC-13 localization. Indeed, we observe a reduction in endogenous UNC-13 levels in *rimb-1* mutants consistent with this model. However, the observed reduction in RIMB-1 cannot fully account for the extent of UNC-13 loss in *cla-1* mutants. Two lines of evidence support this conclusion. First, *cla-1(ΔL)* single mutants have wild-type RIMB-1 levels but exhibit reduced UNC-13 levels. Second, *cla-1(ΔS/M/L);unc-10* exacerbates loss of UNC-13 beyond that of *unc-10* alone, whereas *rimb-1;unc-10* does not. Though these findings support RIMB-1-dependent and independent roles for CLA-1 in regulating the synaptic localization of UNC-13, the mechanism of the latter remains to be elucidated.

We found that *cla-1(ΔL)*, *cla-1(ΔS/M/L)* and *unc-10* single and double mutants dramatically reduced UNC-13L fluorescence intensity, whereas UNC-13S was only reduced in *cla-1(ΔL)* and *cla-1(ΔS/M/L)* mutants. How might this contribute to the severity of the neurotransmission defect in the single and double *cla-1* and *unc-10* mutants? While UNC-13L and UNC-13S share a common C-terminal MUN domain required for priming, the N-terminal unique to UNC-13L is responsible for its enrichment at the AZ through interactions with UNC-10 (3, 42–44). UNC-13L supports a fast component of release that is EGTA-resistant, whereas UNC-13S-dependent release is slower and EGTA-sensitive (20, 45). Similar traits have been observed in *Drosophila* Unc13A and B as well as murine Munc13-2/3 (long and short isoforms, respectively), indicating these differential release kinetics are highly conserved (46, 47). In *C. elegans,* these distinct functional properties are thought to arise in part from UNC-10-dependent interactions that relieve UNC-13L inhibitory homodimerization and stabilize the isoform in close proximity to UNC-2 Ca^2+^ channels (20, 40). This is similar to mammalian synapses in which the N-terminal region of RIM interacts with ubMunc13-2 to disrupt autoinhibition (28). Thus, in the absence of UNC-10, combined loss of UNC-13L and UNC-2 localization can account for the reduced Ca^2+^-sensitivity of evoked release in *unc-10* mutants (10, 11, 17, 19).

Since UNC-13L is dramatically reduced in both *cla-1(ΔL);unc-10* and *cla-1(ΔS/M/L);unc-10*, the persistence of docked and primed SVs in these double mutants is attributable to UNC-13S, as the *unc-13 null* mutants lack primed SVs (3). The severe release defects of *cla-1;unc-10* double mutants indicate that UNC-13S is unable to support release downstream of priming in the near absence of UNC-13L. This is consistent with UNC-13L-specific mutants that phenocopy the *cla-1;unc-10* double mutants (3, 20). Likewise in *Drosophila*, Unc13B alone (46), or expression of an Unc13A C-terminal fragment, was unable to fully support high-probability evoked release at NMJs downstream of SV docking (48). Both *cla-1(ΔL)* and *cla-1(ΔS/M/L)* mutants have relatively mild synaptic defects compared to *unc-10* mutants despite a significant reduction in UNC-13 puncta number. Our data suggest that this release in *cla-1* mutants is supported by the continued presence of UNC-10. The decrease in charge integral observed in *cla-1(ΔL)* and *cla-1(ΔS/M/L)* mutants is most likely attributable to reduced UNC-13S levels.

In summary, using endogenously tagged synaptic proteins, functional analysis and EM, this study places CLA-1 and UNC-10 as upstream regulators of the *C. elegans* synaptic components UNC-2, RIMB-1 and UNC-13. While CLA-1 plays a minor supporting role in UNC-2 localization in conjunction with UNC-10, its impact on UNC-13L appears to be the predominant role of CLA-1 in synaptic function. This has overlapping design principles with other model organisms (RIM/RBP and RIM/ELKS in mouse and Fife/RIM and BRP/RBP in *Drosophila*) (18, 49) and supports an evolutionarily conserved arrangement of AZ scaffolding proteins that are necessary for activation and localization of the fusion machinery within nanodomains for precise coupling to Ca^2+^ channels (48, 50).

## Materials and Methods

### CRISPR/Cas9 Genome Editing.

#### Deletion of the CLA-1S isoform.

To generate the *cla-1(kur5)* short isoform deletion allele, we used two guide RNAs to remove the intronic sequence between exons 32 and 33 of the cla-1a/long isoform, which serves as a promoter for the short isoform of cla-1. The resulting fusion can only express the M/L isoforms of cla-1. Guide RNAs were synthesized as ALT-R CRISPR (cr)RNAs and injected at 1.5 μM in a mix with 1.5 μM ALT-R Cas9 protein, 1.5 μM ALT-R tracRNA, 6 μM Ultramer DNA oligo repair template (197 bp), and cocrispr reagents including 1.5 μM dpy-10 crRNA and 6 μM dpy-10(cn64) repair template (51). All guide RNAs, DNA oligos, and Cas9 protein were synthesized by Integrated DNA Technologies. The guide RNA sequences were: TAAATGGCTTCAGAATATTG and GTCACACTTTTCCAGGATGA. The resulting deletion was flanked by the regions: 5′: AAATGGCTTCAGAATATTGAA 3′: GATGATGGAAACCTTGCCAA.

#### Endogenous GFP tagging of UNC-13.

To generate endogenously tagged *unc-13*::GFP strain, we injected a mixture of a *unc-13*-specific crRNA (ATACGAGACAGAAACAGTAA), a tracrRNA, a homologous repair construct, and Cas9 into the gonads of wild-type N2 animals. The repair construct was built by subcloning the 1.5 kb upstream and 700 bp downstream sequences from the *unc-13* 3′ end into the 5′ and 3′ ends of GFP in frame, respectively. Co-CRISPR (*dpy-10*) reagents were also included in the injection mixture. Progeny of F1 Roller animals were screened by PCR for GFP insertion.

### RT-PCR Validation of cla-1(S) Expression.

mRNA expression of the long and medium isoforms of cla-1 in the *cla-1(S)* deletion mutant were assessed through RT-PCR of complementary DNA (cDNA) from these mutants and compared with wild-type N2 levels (Details in *SI Appendix*, *Methods*).

#### Thrashing Assays.

Thrashes from a single worm after 1-min equilibration in M9 solution were scored as number of completed thrashing waves/min measured at the head (a wave equals a head turn to one side and back again). Prism (GraphPad) was used to graph data and for statistical analysis. (Details in *SI Appendix*, *Methods*).

### Electrophysiology.

Electrophysiological methods were as previously described (15). Data were acquired using Pulse software (HEKA) run on a Dell computer. Subsequent analysis and graphing were performed using Pulsefit (HEKA), Mini Analysis (Synaptosoft Inc), and Igor Pro (Wavemetrics) (Details in *SI Appendix*, *Methods*).

### Electron Microscopy.

Strains were prepared using HPF fixation and freeze substitution (FS) as previously described (8, 9). (Details in *SI Appendix*, *Methods*).

### Fluorescence Microscopy.

#### Image acquisition and quantification for endogenously tagged UNC-2 and AZ proteins.

Fluorescent microscopy was performed as described previously (17). Images were obtained with a 63×/1.4 numerical aperture on a Zeiss Axio-Observer Z1 microscope, captured using a Zyla 4.2 PLUS (Andor) with Spectra X solid-state light engine (Lumencor) as light source. With the exception of UNC-13 data, a line-scanning method (Metamorph) was utilized to quantify the maximal projection images to produce the average peak fluorescent intensity. (Details in *SI Appendix*, *Methods*).

#### Confocal acquisition of fluorescently tagged RIMB-1::Skylan-S.

Confocal imaging of fluorescently-tagged RIMB-1::Skylan-S were collected from L4 animals anesthetized with 10 mM levamisole in M9 buffer. using a Zeiss Axio Observer Z1 microscope stage outfitted with a Plan-Apochromat 63× 1.4 NA objective and a Yokogawa CSU-W1 spinning disk confocal scanner attached to a Prime 95B camera. Maximum intensity projections of z-stack images were cropped and straightened around the dorsal nerve cord (DNC) using ImageJ/Fiji, and were analyzed using a Matlab script designed to identify puncta in individual TIFF files based on local means thresholding coupled with watershed segmentation. (Details in *SI Appendix*, *Methods*).

#### Confocal acquisition of UNC-13 isoforms under the pUNC-129 promoter.

Young adult worms (~10) were imaged on an Olympus Fluoview FV10i inverted laser scanning confocal microscope with the 60× (NA 1.35) oil immersion lens and optical zooming to a total magnification of 120×. Fluorescent analysis was conducted using NIH FIJI/ImageJ software in which max projections were created from obtained z-stacks; DNCs were straightened. Fluorescent levels and peaks from a 40-µm line along the nerve cord were identified from the Plot Profile data using peak finder in Matlab. Statistical analysis was conducted in Prism (GraphPad) using one-way ANOVA with Dunnett’s multiple comparison test. (Details in *SI Appendix*, *Methods*).

A detailed strain list is included in *SI Appendix*, Table S1.

## Supplementary Material

Appendix 01 (PDF)

## Data Availability

All study data are included in the article and/or *SI Appendix*. All reagents are available upon request.
