# Supplementary material for: C. elegans Clarinet/CLA-1 recruits RIMB-1/RIM-binding protein and UNC-13 to orchestrate presynaptic neurotransmitter release
Source: Proc Natl Acad Sci U S A. 2023 May 15;120(21):e2220856120. doi: 10.1073/pnas.2220856120 (PMC10214197; doi:10.1073/pnas.2220856120)
Supplement: Supplementary file 1 — Appendix 01 (PDF) [file pnas.2220856120.sapp.pdf]

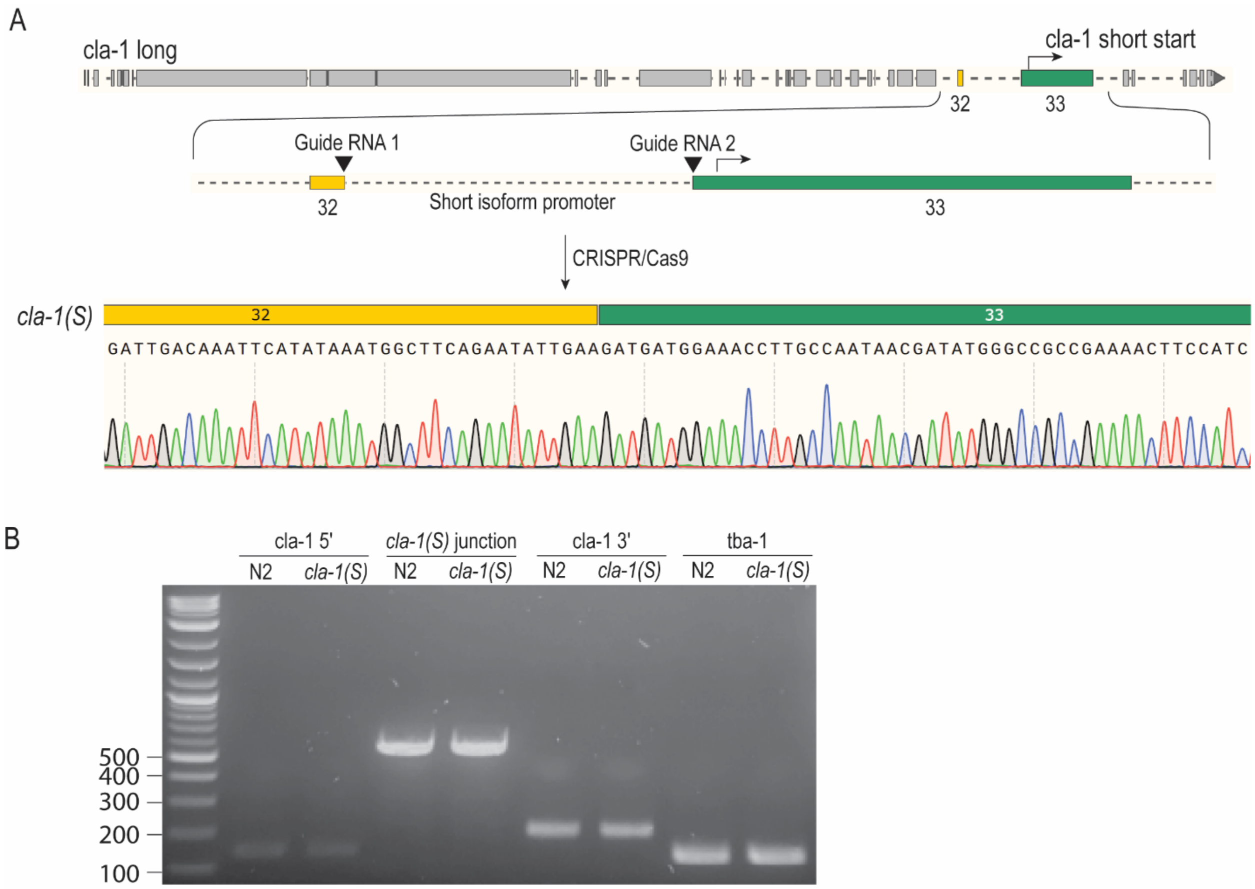

S1. Generation and validation of *cla-1(S)* deletion allele. (A) The *cla-1(S)* allele was generated through CRISPR-mediated deletion of the promoter region of the short isoform of *cla-1* in the intronic region between exons 32 and 33 of the *cla-1a/*long isoform. The entire intron was removed, and sequencing of genomic DNA generated from the *cla-1(S)* allele revealed a clean fusion between exons 32 and 33. **(B)** *cla-1* long and medium isoform mRNA is expressed in the *cla-1(S)* allele at similar levels as wildtype N2 animals, as demonstrated by PCR of multiple regions of *cla-1* from cDNA, including the 5′ region of the *cla-1a* long isoform, across the junction between *cla-1a* exons 32 and 33 (the location of the intron deletion), and the *cla-1* 3′ PDZ domain. Wildtype N2 and *cla-1(S)* cDNA sample loads were controlled with *tba-1*/tubulin alpha, which was also expressed at similar levels between the two genotypes.

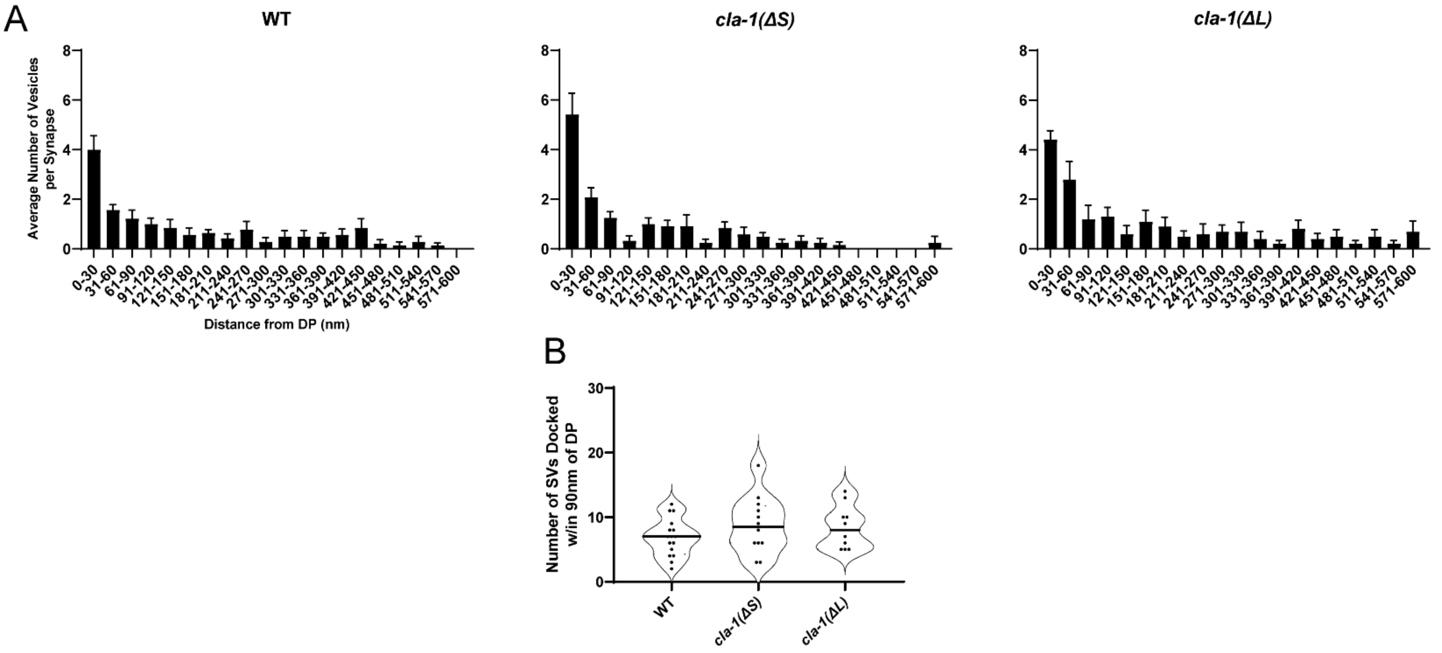

S2. The distribution of docked SVs from the DP is similar to that of WT in both the *cla-1(ΔS)* and *cla-1(ΔL)* isoform mutants. (A) The distribution of docked vesicles as measured from the DP are unchanged in either *cla-1(ΔS)* or *cla-1(ΔL)* single isoform mutants. **(B)** Though the proximal vesicle pool (SVs docked within 90nm of the DP) trends higher in the *cla-1(ΔS)* and *cla-1(ΔL)* mutants there is no difference from the WT. One way ANOVA.

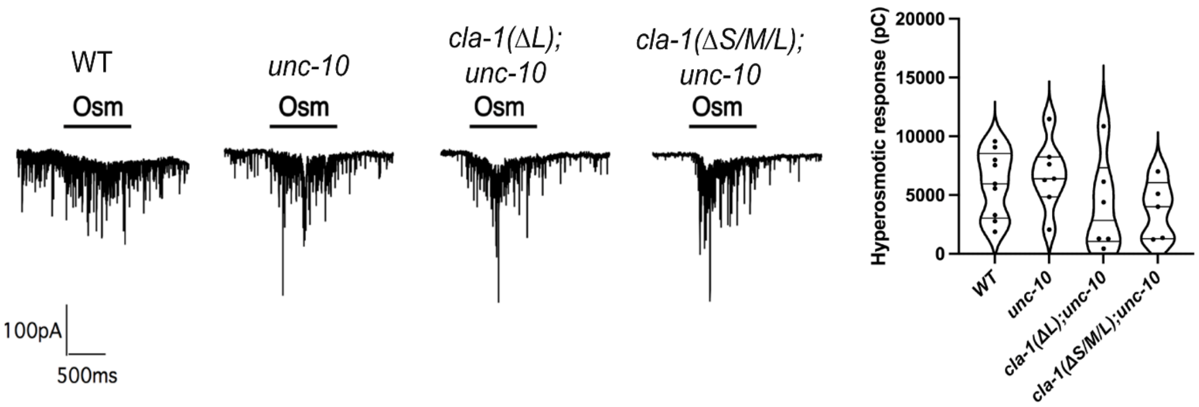

S3. Application of hypertonic solution reveals that morphologically docked vesicles in *unc-10* and *cla-1* double mutants are fusion competent. Neither the single *unc-10* nor the *cla-1(ΔS/M/L)* and *cla-1(ΔL)* double mutants exhibit a significant reduction in the primed, fusion-competent vesicle pool.

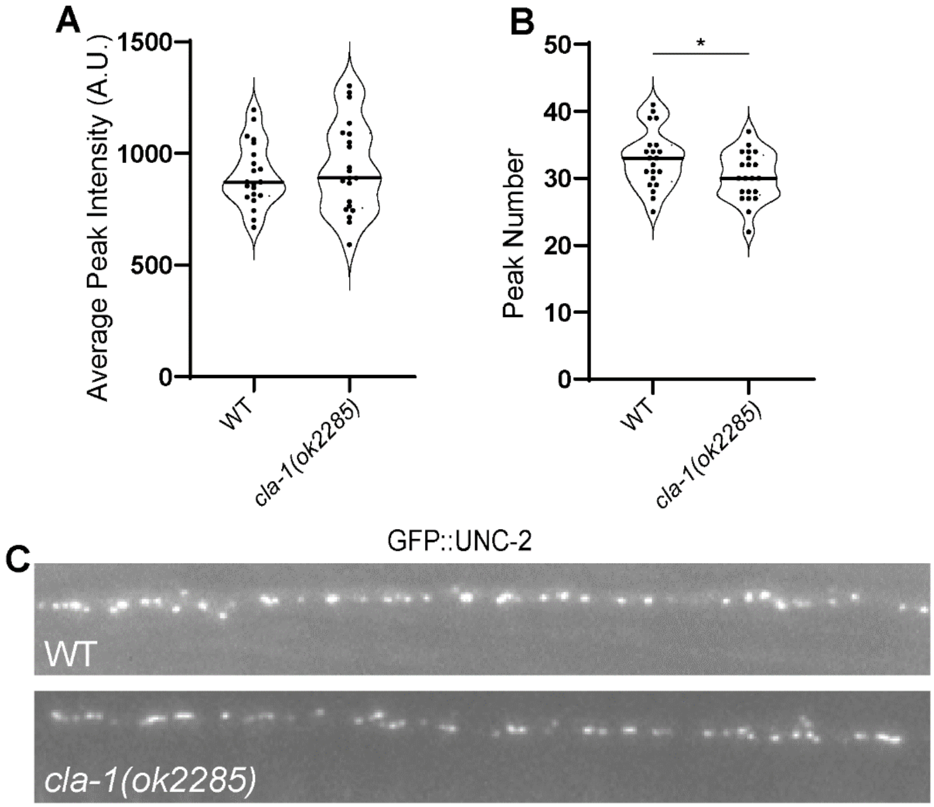

S4. A second *cla-1 null* allele exhibits a minor reduction in UNC-2 peak number. Though **(A)** the average peak intensity is not different in *cla-1(ok2285), a second cla-1 null* allele, **(B)** there was a minor reduction in peak number of UNC-2 similar to that seen in *cla-1(wy1048(ΔS/M/L))* mutant animals. T-test *p < 0.05 (C) Representative images of the dorsal nerve cords in wild-type and *cla-1(ok2285)* animals.

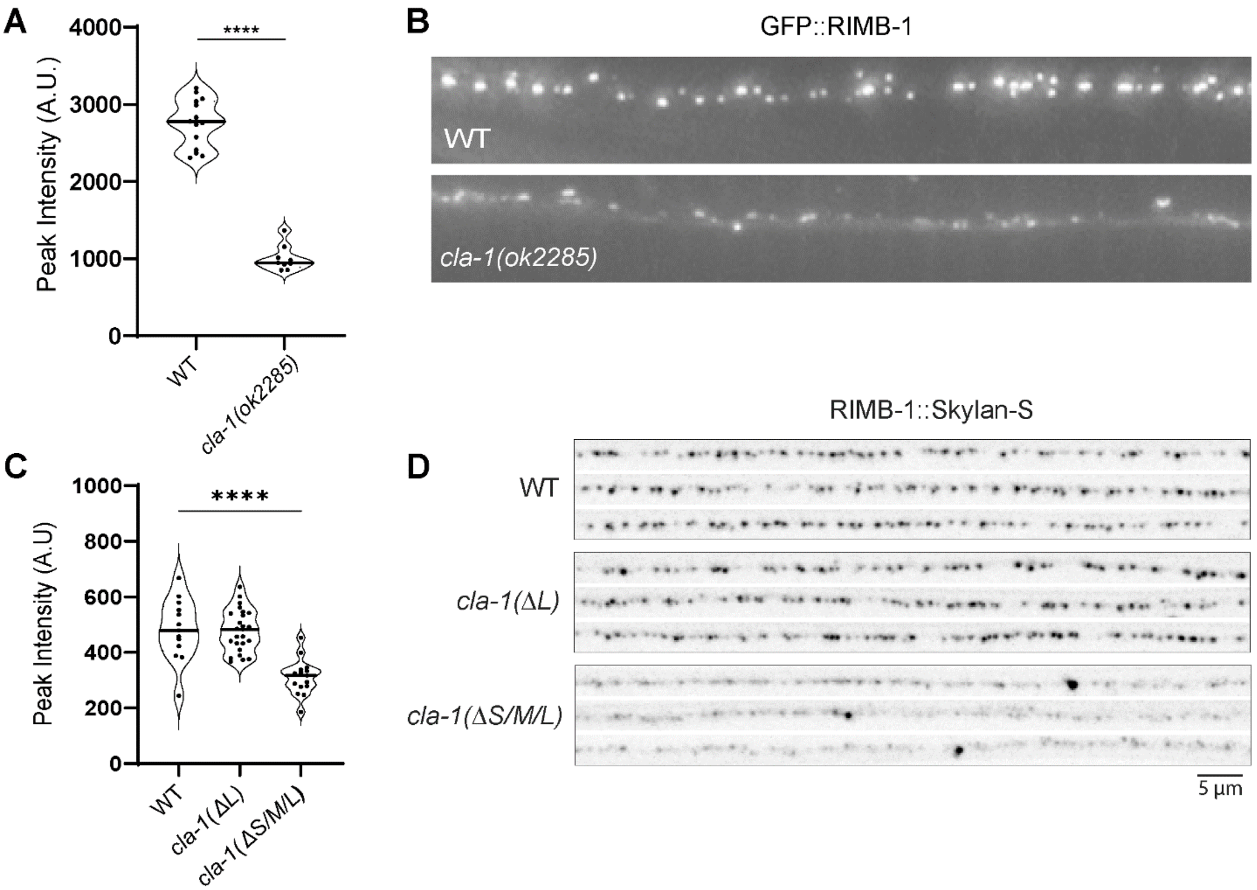

S5. RIMB-1 is reduced in *cla-1 null* mutants. (A-B). The *cla-1(ok2285)* mutant exhibits a similar reduction in levels of RIMB-1 to that seen in a *cla-1(wy1048(ΔS/M/L))* mutant. Student's t-test. ****p < 0.0001 **(C-D)** The reduction in RIMB-1 levels seen in *cla-1 null* mutants, but not in *cla-1(ΔL),* using a N-terminal RIMB-1 GFP tag, were verified using a C-terminal RIMB-1 SKYLAN tag. One way ANOVA. ****p < 0.0001

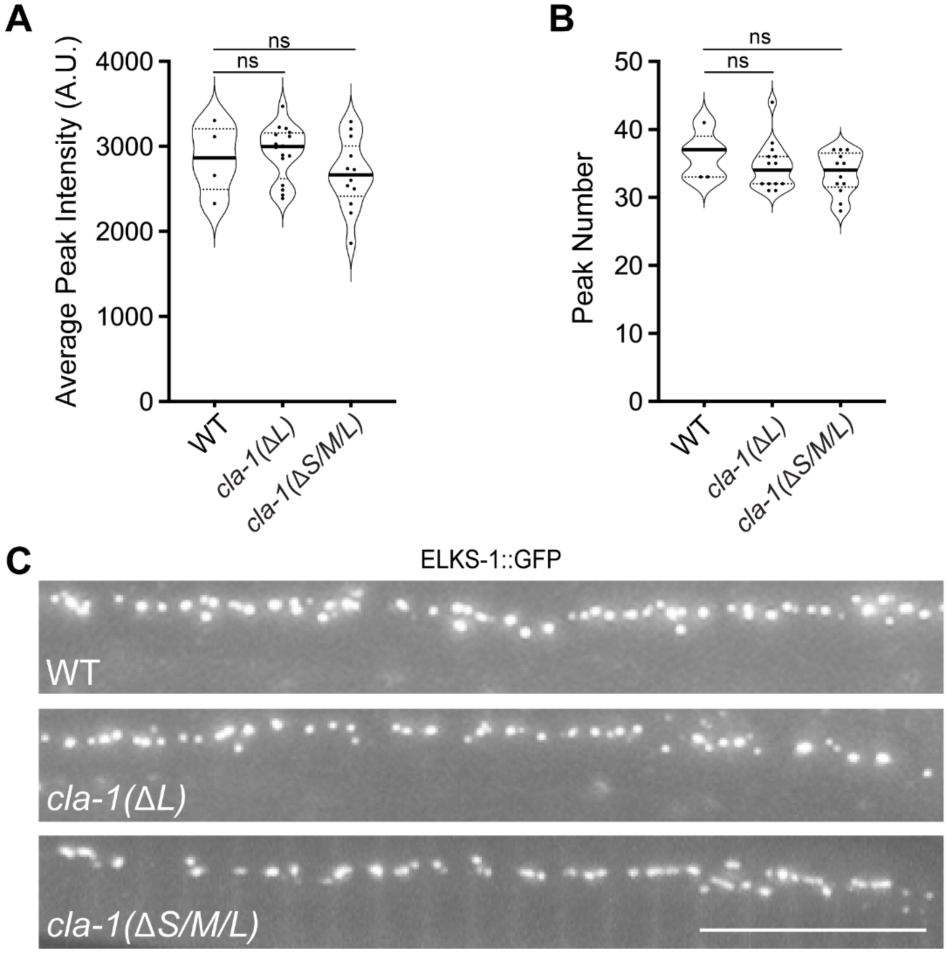

S6. Synaptic localization of ELKS-1/ELKS is not altered in *cla-1* mutants. The average peak intensity (A) and peak number (B) of ELKS-1, an active zone protein, were not significantly different between wild-type and *cla-1(ΔL),* and *cla-1(ΔS/M/L)* mutant animals. ns, not significant, One-way ANOVA, Tukey's analysis. (C) Representative images of the dorsal nerve cords in wild-type and *cla-1* animals. The length of the images is 40 μm. Scale bar, 10 μm.

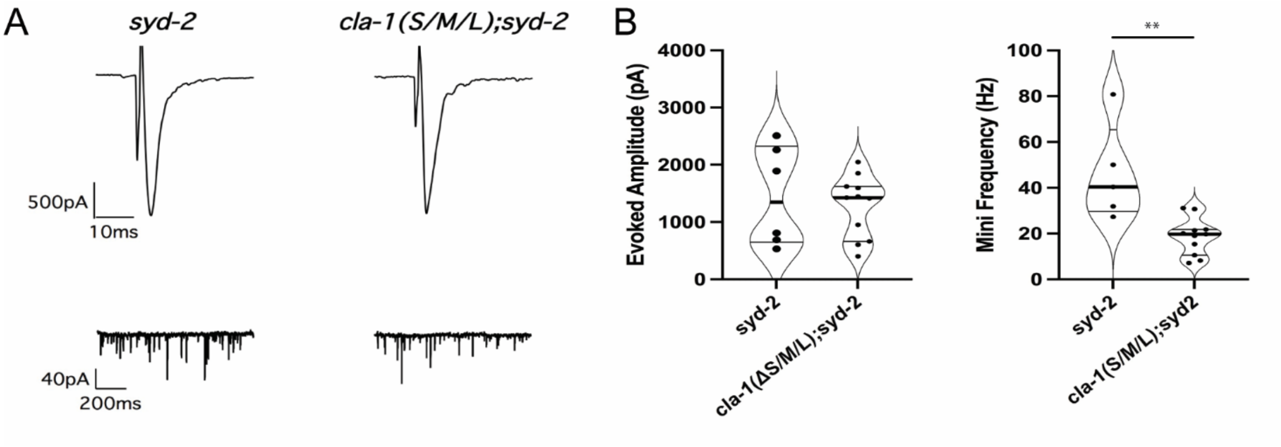

S7. SYD-2-independent roles for CLA-1 in the regulation of endogenous release. (A) Representative traces of evoked current amplitude in whole-cell voltage-clamped post-synaptic muscles in *cla-1(ΔS/M/L);syd-2* double mutants is unchanged relative to syd-2 (upper traces). Lower traces of endogenous minis in *cla-1(ΔS/M/L);syd-2* double mutants demonstrate a significant decrease in frequency relative to *syd-2* alone. **(B)** Quantification of the electrophysiological data. Student's t-test **p <0.01.

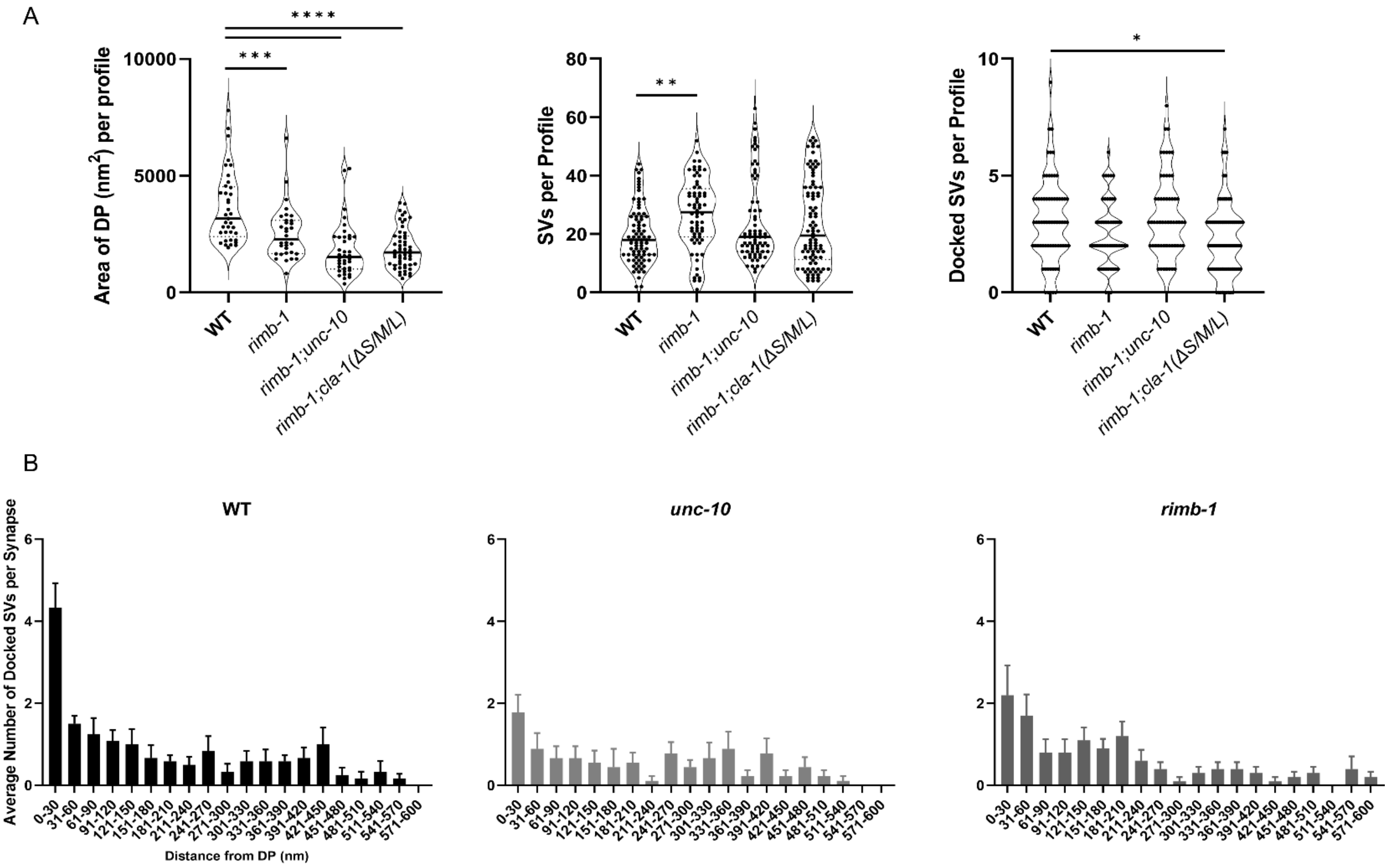

S8. *rimb-1* mutants have a reduction in DP area as observed by EM. (A) Consistent with observations in *Drosophila* mutants without Rim-binding protein, we observed smaller DPs in all *rimb-1* mutants. Like *unc-10* mutants, *rimb-1* mutants have an increase in synaptic vesicles. Though trending slightly lower, *rimb-1* mutants do not have less docked vesicles than WT. *p< 0.05, **p< 0.01, ***p< 0.001, ****p< 0.0001 **(B)** The *rimb-1* null mutants exhibit a reduction in proximally docked vesicles similar to that observed in *unc-10* null mutants. One way ANOVA, Tukey's post-hoc analysis.

Table S1. List of strains used in this study.

Strain name	Genotype	Source
HKK845	unc-2(cim104[gfp::unc-2])	(1)
HKK1300	rimb-1(ce828);unc-2(cim104[gfp::unc-2])	(1)
HKK1829	cla-1(kur5);unc-2(cim104[gfp::unc-2])	This study
HKK1195	cla-1(ok560);unc-2(cim104[gfp::unc-2])	This study
HKK1257	cla-1(wy1048);unc-2(cim104[gfp::unc-2])	This study
HKK1149	unc-10(md1117);unc-2(cim104[gfp::unc-2])	(1)
HKK1506	rimb-1(ce828);unc-10(md1117);unc-2(cim104[gfp::unc-2])	(1)
HKK1972	cla-1(kur5);unc-10(md1117);unc-2(cim104[gfp::unc-2])	This study
HKK1259	cla-1(ok560);unc-10(md1117);unc-2(cim104[gfp::unc-2])	This study
HKK1249	cla-1(wy1048);unc-10(md1117);unc-2(cim104[gfp::unc-2])	This study
HKK1327	rimb-1(cim116[gfp::rimb-1])	(1)
HKK1351	unc-10(md1117);rimb-1(cim116[gfp::rimb-1])	(1)
HKK1708	cla-1(kur5);rimb-1(cim116[gfp::rimb-1])	This study
HKK1366	cla-1(ok560);rimb-1(cim116[gfp::rimb-1])	This study
HKK1350	cla-1(wy1048);rimb-1(cim116[gfp::rimb-1])	This study
HKK1831	cla-1(kur5);unc-10(md1117);rimb-1(cim116[gfp::rimb-1])	This study
HKK1830	cla-1(ok560);unc-10(md1117);rimb-1(cim116[gfp::rimb-1])	This study
HKK1832	cla-1(wy1048);unc-10(md1117);rimb-1(cim116[gfp::rimb-1])	This study
HKK1620	unc-13(cim127[unc-13::gfp])	This study
HKK1973	cla-1(kur5);unc-13(cim127[unc-13::gfp])	This study
HKK1694	cla-1(ok560);unc-13(cim127[unc-13::gfp])	This study
HKK1697	cla-1(wy1048);unc-13(cim127[unc-13::gfp])	This study
HKK1625	unc-10(md1117);unc-13(cim137[unc-13::gfp])	This study
HKK1654	rimb-1(ce828);unc-13(cim137[unc-13::gfp])	This study
HKK1783	cla-1(ok560);unc-10(md1117);unc-13(cim127[unc-13::gfp])	This study
HKK1784	cla-1(wy1048);unc-10(md1117);unc-13(cim127[unc-13::gfp])	This study
HKK1781	rimb-1(ce828);unc-10(md1117);unc-13(cim127[unc-13::gfp])	This study
HKK1780	rimb-1(ce828);cla-1(ok560);unc-13(cim127[unc-13::gfp])	This study
HKK1782	rimb-1(ce828);cla-1(wy1048);unc-13(cim127[unc-13::gfp])	This study
HKK1528	elks-1(cim121[elks-1::gfp])	(1)
HKK1545	cla-1(ok560);elks-1(cim121[elks-1::gfp])	This study
HKK1544	cla-1(wy1048);elks-1(cim121[elks-1::gfp])	This study
HKK1585	rimb-1(ce828);unc-10(md1117)	This study
HKK1578	rimb-1(ce828);cla-1(wy1048)	This study
HKK1494	syd-2(ok217);cla-1(wy1048)	This study
HKK1576	cla-1(ok560);rimb-1(ce828)	This study
HKK1589	unc-10(md1117)	(1)
TV24709	rimb-1(ox704[skylan-S]);Unc-2(ox672[HALO])	(2)
TV24679	rimb-1(ox704[skylan-S]); cla-1(wy1048); unc-2(ox672[HALO])	This study
TV24680	rimb-1(ox704); cla-1(ok560[skylan-S]); unc-2(ox672[HALO])	This study
KP6897	nuIs486 punc-13::UNC-13L::mCherry	(3)
SY1798	cla-1(ok560);punc-129::UNC-13L::mCherry	This study
SY1797	cla-1(wy1048);punc-129::UNC-13L::mCherry	This study
SY1799	unc-10(md1117);punc-129::UNC-13L::mCherry	This study

SY1781	cla-1(ok560);unc-10(md117);punc-129::UNC-13L::mCherry	This study
SY1780	cla-1(wy1048);unc-10(md1117)punc-129::UNC-13L::mCherry	This study
KP7285	nuIs497 punc-13::UNC-13S::mCherry	(3)
SY1782	cla-1(ok560);punc-129::UNC-13S::mCherry	This study
SY1794	cla-1(wy1048);punc-129::UNC-13S::mCherry	This study
SY1793	unc-10(md1117);punc-129::UNC-13S::mCherry	This study
SY1796	cla-1(ok560);unc-10(md117);punc-129::UNC-13S::mCherry	This study
SY1795	cla-1(wy1048);unc-10(md1117)punc-129::UNC-13S::mCherry	This study
PTK35	cla-1(kur5)	This study
RB778	cla-1(ok560)	(4)
SY1685	cla-1(wy1048)	This study
PTK40	cla-1(kur5);unc-10(md1117)	This study
SY1687	cla-1(ok560);unc-10(md1117)	This study
SY1686	cla-1(wy1048);unc-10(md1117)	This study

Supplemental Methods

RT-PCR Validation of *cla-1(S) expression*

RNA was isolated from N2 and *cla-1(S)* mutants by grinding in liquid nitrogen using a mortar and pestle, followed by homogenization using Qiashredder Columns (Qiagen), and then finally purified with the RNeasy Plus Mini Kit (Qiagen). RNA was reverse transcribed to cDNA using the iScript cDNA Synthesis Kit (Bio-Rad). Primer sets were designed to amplify a 5′ region of the cla-1a long isoform, across the junction between cla-1a exons 32 and 33 (the location of the intron deletion), and the cla-1 3′ PDZ domain. PCR of these sites from cDNA was performed using a fixed series of cycles (22, 26, and 29 cycles) and cla-1 long and medium isoform mRNA was expressed in the *cla-1(S)* allele at similar levels as wildtype N2 animals, with amplicon bands appearing at the same cycle number as the appearance of N2 bands.

Primers sequenced used in RT-PCR include:

cla-1 5′: TCAGTAAAGGTCCACCTCTGG and TGCTCGTCGAAGCACATCTT

cla-1(S) junction: TTCCCAATGCCTCCAACTGC and GAAGAATACGAAGCACCAGC

cla-1 3′: TGACTCGCAGTTACAAGCATC and TGTTCACAATTCGTTCCACCTC

tba-1: GTACACTCCACTGATCTCTGCTGACAAG and CTCTGTACAAGAGGCAAACAGCCATG

RNA was reverse transcribed to cDNA using the iScript cDNA Synthesis Kit (Bio-Rad). Primer sets were designed to amplify a 5′ region of the cla-1a long isoform, across the junction between cla-1a exons 32 and 33 (the location of the intron deletion), and the cla-1 3′ PDZ domain. PCR of these sites from cDNA was performed using a fixed series of cycles (22, 26,

and 29 cycles) and cla-1 long and medium isoform mRNA was expressed in the *cla-1(S)* allele at similar levels as wildtype N2 animals, with amplicon bands appearing at the same cycle number as the appearance of N2 bands.

Primers sequenced used in RT-PCR include:

cla-1 5′: TCAGTAAAGGTCCACCTCTGG and TGCTCGTCGAAGCACATCTT

cla-1(S) junction: TTCCCAATGCCTCCAACTGC and GAAGAATACGAAGCACCAGC

cla-1 3′: TGACTCGCAGTTACAAGCATC and TGTTCACAATTCGTTCCACCTC

tba-1: GTACACTCCACTGATCTCTGCTGACAAG and
CTCTGTACAAGAGGCAAACAGCCATG

Electrophysiology

Animals were immobilized with cyanoacrylic glue, a lateral cuticle incision was made, and internal organs were removed to expose the ventral neuromuscular junctions. Muscle recordings were made in the whole-cell voltage-clamp configuration (holding potential –60 mV) using a HEKA EPC-10 patch-clamp amplifier and digitized at 2.9 kHz. The extracellular solution contained (in mM): NaCl 150; KCl 5; $CaCl_2$ 5; $MgCl_2$ 4, glucose 10; sucrose 5; HEPES 15 (~340mOsm, pH 7.3). The patch pipette intracellular contained (in mM): KCl 120; KOH 20; $MgCl_2$ 4; (*N*-tris[Hydroxymethyl] methyl-2-aminoethane-sulfonic acid) 5; $CaCl_2$ 0.25; Na^2ATP 4; sucrose 36; EGTA 5 (pH 7.2, ~315mOsm). Hyperosmotic data were recorded using a 1sec pressure-ejected pulse of 840 mOsm extracellular solution achieved through addition of sucrose.

Electron Microscopy

Twenty to thirty young adult worms were placed in specimen chambers filled with *E. coli* and frozen at −180°C, using liquid nitrogen under high pressure (Leica HPM 100, Oberkochen, Germany). Samples then underwent freeze substitution (Reichert AFS, Leica) using the following program: −90°C for 107 hours with 0.1% tannic acid followed by 2% OsO_4 in anhydrous acetone, incrementally warmed at a rate of 5°C/hour to −20°C, and kept at −20°C for 14 hours before increasing temperature by 10°C/hour to 20°C; samples were then infiltrated with 50% Epon/acetone for 4 hours, 90% Epon/acetone for 18 hours, and 100% Epon for 5 hours; finally, samples were embedded in Epon and incubated for 48 hours at 65°C (5). Ultra-thin (40 nm) serial sections were acquired using an Ultracut 6 (Leica) and collected on formvar-covered, carbon-coated copper grids (EMS, FCF2010-Cu). Sections were post-stained with 2.5% aqueous uranyl acetate for 4 minutes, followed by Reynolds lead citrate for 2 minutes (5). Images were obtained using either a JEOL JEM1220 or JEM-1400F transmission electron microscope, operating at 80 kV. Micrographs were acquired using one of the following cameras: Gatan Es1000W 11MP CCD, AMT NanoSprint1200-S CMOS or BioSprint 12M-B CCD Camera with AMT software (Version 7.01). Cholinergic synapses at the NMJ of the ventral nerve cord were identified based on established synaptic morphology (6). Sections containing a DP, as well as two flanking sections on either side of the DP, were analyzed blinded to genotype using NIH FIJI/ImageJ software. SVs were counted as docked when the SV membrane was fully contacting the plasma membrane of the neuron terminal (distance = 0 nm), SVs that were within 1–5 nm of the plasma membrane that exhibited small tethers were not scored as docked. The distribution of docked SVs from the DP was calculated for each section containing a DP, as well as one section on either side, using the ROI data from FIJI with Matlab scripts written by the Watanabe and Jorgensen labs (7). Values were imported to Prism (GraphPad) for statistical analysis using One-way ANOVA with Tukey post hoc analysis, or Kruskal-Wallis with Dunn's

test, for multiple comparisons. An unpaired t-test was used when comparing only two genotypes.

Fluorescence Microscopy

Image acquisition and quantification for endogenously tagged UNC-2 and AZ proteins.

Fluorescent microscopy was performed as described previously (1). All microscopy was performed on adult animals 20-22 hours post L4 stage. Animals were transferred onto a 2% agarose gel pad with 6 mM levamisole solution in M9 buffer for immobilization. To ensure complete immobilization, animals were incubated in solution for 10 minutes before imaging. The dorsal nerve cord located at the posterior gonad arm was selected as the image area, as it consists of specific GABAergic and cholinergic presynaptic terminals. This area comprises of minimal autofluorescence and lacks dendrites and cell bodies that may interfere with analysis. Images were obtained on a 63x/1.4 numerical aperture on a Zeiss Axio-Observer Z1 microscope. Images were captured using a Zyla 4.2 PLUS (Andor) with Spectra X solid-state light engine (Lumencor) as light source. For a given GFP transgenic animal, the same light intensity and exposure time setting were used. While not all animals were imaged simultaneously, each strain was imaged with its respective control to ensure consistency among image quality and quantification. Variations in intensity or peak number within the same genotype between different imaging sessions were negligible. Images were obtained as horizontal slices or Z-stacks, and maximal projection was applied and used for quantification. With the exception of UNC-13 data, a line-scanning method (Metamorph) was utilized to quantify the maximal projection images to produce the average peak fluorescent intensity. Pixel intensity of 282 pixel length or 30 microns were measured in each image. To obtain the true peak intensity of each puncta, the average background intensity from an adjacent area was also measured and subtracted. Peaks above a threshold value were counted as a true peaks. The

threshold was arbitrarily set to exclude small background fluctuations, and this same threshold value was applied to all the images of a given transgenic line. Since UNC-13 images are comprised of discrete puncta and surrounding diffuse signals adaptive thresholding was used on selected 282 x 47 pixel areas from maximum projection images to obtain a series of puncta from which maximal puncta intensity values and puncta numbers were using Integrative Morphometry Analysis (Metamorph). For clear separation of these two methods we used peak number/average intensity for the linescan method and puncta number/max intensity for adaptive thresholding.

Confocal acquisition of UNC-13 isoforms under the pUNC-129 promoter

Young adult worms (~10) were placed in a drop of M9 on 2% agarose pads (in M9 buffer containing 10 mM Sodium Azide (NaN_3)) on a glass slide under a coverslip. Images of dorsal nerve cords were collected on an Olympus Fluoview FV10i inverted laser scanning confocal microscope with the 60X (NA 1.35) oil immersion lens and optical zooming to a total magnification of 120X. The same imaging parameters were used for each genetic background of the same fluorescence marker. Control strains were always imaged on the same day as mutant strains. Fluorescent analysis was conducted using NIH FIJI/ImageJ software in which max projections were created from obtained z-stacks, nerve cords were straightened, and all images were subjected to background subtraction with a rolling ball radius of 50. Fluorescent levels were extracted from a 40μm line along the nerve cord and peaks were identified from the Plot Profile data using peak finder in Matlab with an arbitrary threshold set to account for background fluctuations which was applied to all images for a given fluorescent marker. Statistical analysis was conducted in Prism (GraphPad) using one-way ANOVA with Dunnett’s multiple comparison test.

References

1. K. H. Oh, M. D. Krout, J. E. Richmond, H. Kim, UNC-2 CaV2 Channel Localization at Presynaptic Active Zones Depends on UNC-10/RIM and SYD-2/Liprin-α in Caenorhabditis elegans. *Journal of Neuroscience* **41**, 4782–4794 (2021).
2. P. T. Kurshan, *et al.*, γ-Neurexin and Frizzled Mediate Parallel Synapse Assembly Pathways Antagonized by Receptor Endocytosis. *Neuron* **100**, 150-166.e4 (2018).
3. Z. Hu, X. J. Tong, J. M. Kaplan, UNC-13L, UNC-13S, and Tomosyn form a protein code for fast and slow neurotransmitter release in Caenorhabditis elegans. *Elife* **2013** (2013).
4. Z. Xuan, *et al.*, Clarinet (CLA-1), a novel active zone protein required for synaptic vesicle clustering and release. *Elife* **6** (2017).
5. H. Liu, *et al.*, Protocols for electrophysiological recordings and electron microscopy at C. elegans neuromuscular junction. *STAR Protoc* **2**, 100749 (2021).
6. J. G. White, E. Southgate, J. N. Thomson, S. Brenner, The Structure of the Nervous System of the Nematode Caenorhabditis elegans. *Philosophical Transactions of the Royal Society B: Biological Sciences* **314**, 1–340 (1986).
7. S. Watanabe, M. W. Davis, G. F. Kusick, J. Iwasa, E. M. Jorgensen, SynapsEM: Computer-Assisted Synapse Morphometry. *Front Synaptic Neurosci* **12** (2020).
